# Platelet-derived exosomes promote neutrophil extracellular trap formation during septic shock

**DOI:** 10.1186/s13054-020-03082-3

**Published:** 2020-06-29

**Authors:** Yang Jiao, Weiwei Li, Wei Wang, Xingyu Tong, Ran Xia, Jie Fan, Jianer Du, Chengmi Zhang, Xueyin Shi

**Affiliations:** 1 Department of Anesthesiology and Intensive Care Unit, Xinhua Hospital, School of Medicine, Shanghai Jiaotong University, 1665 Kongjiang Road, Shanghai, 200092 China; 2 Department of Surgery, University of Pittsburgh School of Medicine, Pittsburgh, PA 15213 USA

**Keywords:** Septic shock, Neutrophil extracellular traps, Platelets, Exosomes, HMGB1, miRNAs

## Abstract

**Background:**

Platelets have been demonstrated to be potent activators of neutrophil extracellular trap (NET) formation during sepsis. However, the mediators and molecular pathways involved in human platelet-mediated NET generation remain poorly defined. Circulating plasma exosomes mostly originating from platelets may induce vascular apoptosis and myocardial dysfunction during sepsis; however, their role in NET formation remains unclear. This study aimed to detect whether platelet-derived exosomes could promote NET formation during septic shock and determine the potential mechanisms involved.

**Methods:**

Polymorphonuclear neutrophils (PMNs) were cocultured with exosomes isolated from the plasma of healthy controls and septic shock patients or the supernatant of human platelets stimulated ex vivo with phosphate buffer saline (PBS) or lipopolysaccharide (LPS). A lethal cecal ligation and puncture (CLP) mouse model was used to mimic sepsis in vivo; then, NET formation and molecular pathways were detected.

**Results:**

NET components (dsDNA and MPO-DNA complexes) were significantly increased in response to treatment with septic shock patient-derived exosomes and correlated positively with disease severity and outcome. In the animal CLP model, platelet depletion reduced plasma exosome concentration, NET formation, and lung injury. Mechanistic studies demonstrated that exosomal high-mobility group protein 1 (HMGB1) and/or miR-15b-5p and miR-378a-3p induced NET formation through the Akt/mTOR autophagy pathway. Furthermore, the results suggested that IκB kinase (IKK) controls platelet-derived exosome secretion in septic shock.

**Conclusions:**

Platelet-derived exosomes promote excessive NET formation in sepsis and subsequent organ injury. This finding suggests a previously unidentified role of platelet-derived exosomes in sepsis and may lead to new therapeutic approaches.

## Introduction

Sepsis, defined as life-threatening organ dysfunction caused by a dysregulated host response to infection, is a major public health concern [1]. The in-hospital mortality rate of sepsis is recorded as over 10% worldwide, whereas that of septic shock is greater than 40% [2]. Multiple organ dysfunction, including acute lung injury (ALI), is a common manifestation of sepsis. Sepsis is characterized by excessive inflammation in response to infection; however, the molecular mechanisms remain to be fully elucidated [3].

Polymorphonuclear neutrophils (PMNs) are the most abundant leukocytes in mammals, playing crucial roles in innate immune responses during sepsis. In addition to degranulation and phagocytosis, neutrophils also release neutrophil extracellular traps (NETs) to ensnare and kill microbes [4]. NETs are extracellular strands of decondensed DNA decorated with histones and neutrophil granule proteins. However, NETs may function as double-edged swords; overzealous NET formation during sepsis can lead to the development of multiple organ dysfunction [5]. NETs have been found in the lungs and could induce lung endothelial injury mediated by extracellular histones, neutrophil granular proteins, and a tangled web of extracellular DNA [6].

Platelets are not only elements of primary importance in hemostasis and thrombosis, but also essential elements of an integrated inflammatory response [7]. Increasing evidence in vitro and in vivo indicates that platelets may contribute to the NET formation process [8]. However, the mechanism underlying platelet-mediated NET formation remains unclear [9]. Previous studies demonstrated that circulating plasma exosomes, which are small lipid packages that carry a variety of molecules, mostly originated from platelets, induces inflammation and myocardial dysfunction during sepsis [10–12]. Exosomes serve as vehicles to transport nucleotides, lipids, and proteins between cells in physiological and pathological conditions [13]. Study has shown that platelet-derived exosomes sustain autophagy-associated NET formation in systemic sclerosis [14]. However, whether platelet-derived exosomes play a role in NET formation in sepsis has yet to be addressed.

MicroRNAs (miRNAs) are important modulators of gene expression as they induce messenger RNA degradation or prevent translation [15, 16]. Exosome-contained miRNAs may serve as an important mediator of intercellular communication in sepsis [17]. In addition, recent studies have also shown that human platelet high-mobility group box 1 (HMGB1) induces PMNs autophagy and NET formation [14, 18]. Platelets are a source of HMGB1 protein, which is a ubiquitous nuclear and cytosolic protein [19]. Released circulating HMGB1 mediates lethality in LPS-induced endotoxemia and cecal ligation and puncture (CLP)-induced polymicrobial sepsis mouse models [20]. However, whether platelet-derived exosomal HMGB1 and/or miRNAs could modulate NET formation during septic shock needs to be further addressed.

Therefore, the current study aimed to test the hypothesis that exosomal HMGB1 and/or miRNAs from platelets might induce excessive NET formation in septic shock and subsequent acute lung injury. The results show that in sepsis, IκB kinase controls platelet-derived exosome secretion, which in turn induces NET formation. Exosomal HMGB1 and/or miR-15b-5p and miR-378a-3p regulate NET formation through the Akt/mTOR autophagy pathway in PMNs.

## Materials and methods

### Patients

We recruited 21 patients with early (less than 24 h) diagnosis of septic shock who were admitted to the ICU of Xinhua Hospital, Shanghai Jiaotong University, China, from March 2018 to July 2019 (Table 1). Septic shock was defined as persisting hypotension requiring vasopressors to maintain mean arterial pressure (MAP) ≥ 65 mmHg and a serum lactate level >2 mmol/L (18 mg/dL) despite adequate volume resuscitation [1]. We excluded patients under 18 years old; those with pregnancy, severe anemia, active bleeding, platelet disorders, or chemotherapy; or those using full-dose heparin or any other medications that interfere with platelet function. The enrolled patients had 30 mL blood samples collected from a central venous catheter. Twenty-two healthy volunteers provided blood samples that served as controls. The study was approved by the institutional ethics and review board of Xinhua Hospital, and informed consent was obtained from the patients or their representatives.

**Table 1:** Demographic data of the included patients

Characteristics	Septic shock patients (*N* = 21)
Male sex, *n* (%)	16 (76)
Age, years	71 ± 9
Mortality at 28 days, *n* (%)	5 (24)
Comorbidities, *n* (%)	
Arterial hypertension	9 (43)
Diabetes mellitus	2 (9)
Others	10 (48)
Source of sepsis, *n* (%)	
Urinary	3 (14)
Abdominal	18 (86)
Hemodynamic data	
Heart rate/min	100 ± 24
Mean arterial pressure, mmHg	81 ± 13
Norepinephrine dosage, μg/kg/min	0.21 (0.18–0.64)
Ventilatory data	
Respirate rate/min	20 ± 7
PaCO_2_ (mm Hg)	31 ± 8
PaO_2_/FIO_2_ (Kpa)	366 ± 112
Use of mechanical ventilation, *n* (%)	16 (76)
Hematologic and inflammatory data	
Neutrophils, 10^9^/L	13.6 ± 8.9
Hemoglobin, g/dL	102.3 ± 19.6
Platelets, 10^9^/L	150.5 ± 77
Lactate, mmol/L	2.5 (2.1–6.1)
CRP, mg/dL	160 (102–160)
Procalcitonin, ng/mL	47 (18.9–100)
Glasgow score	9.8 ± 3.3
SOFA score	9 (9–11)

*CRP* C-reactive protein, *SOFA* Sequential Organ Failure Assessment

Data are expressed as number (%), mean ± SD, or median (25th–75th percentile)

### Exosome isolation and characterization

Exosomes were isolated from the plasma of healthy controls and septic shock patients or from the supernatant of platelets isolated from healthy volunteers stimulated ex vivo using Total Exosome Isolation Reagent (Thermo Fisher Scientific, Waltham, MA, USA). The detailed isolation procedure and the methods used to determine exosomal morphology, size distribution, and surface marker expression are described in Additional file 1: supplementary methods.

### Platelet purification and activation

Human platelets were isolated from EDTA-anticoagulated venous blood as previously described [21]. Platelet activation was induced upon incubation with PBS or 1 μg/mL LPS (from *Escherichia coli* O111:B4, Sigma) or 0.1 U/mL thrombin for 3 h at 37 °C. More detailed information on all methods is provided in Additional file 1: supplementary methods.

### PMNs isolation and NET induction

PMNs were isolated from the venous blood of healthy volunteers by discontinuous density gradient centrifugation with two commercially available solutions (Histopaque-1077 and Histopaque-1119) of differential density (#10771 and #11191, Sigma; St Louis, MO, USA), according to the manufacturer’s instructions. PMNs were treated with exosomes (100 or 200 μg/mL) up to 14 h or with 50 nM PMA for 3 h at 37 °C as the positive control. To determine the role of ROS and autophagy in NET formation, the following inhibitors and enhancers were used: 3-Methyladenine (3-MA, 5 mM), wortmannin (150 nM), bafilomycin A1 (1 μM), rapamycin (100 nM), or *N*-acetyl-l-cysteine (NAC, 50 μg/mL).

### HL-60 cell culture and transfection

The human acute promyelocytic leukemia cell line HL-60 (ATCC-CCL-240) was maintained in phenol red-free RPMI-1640 medium supplemented with 2 mM L-glutamine, 10% FBS, and 1.25% DMSO for 3 days as described [22]. Cells were then transfected with microRNA control (50 nM), mimics (miR-24-3p, miR-15b-5p, miR-25-3p, miR-126-3p, miR-378a-3p, and miR-155-5p), or inhibitors (miR-15b-5p, miR-378a-3p) (Shanghai GenePharma Co., Ltd., Shanghai, China) for 24–48 h prior to analysis or cocultured with exosomes as per the manufacturer’s instructions.

### Animals

Wild type, male C57BL/6 mice aged 6–8 weeks were purchased from Shanghai Sippr-BK Laboratory Animal Co. Ltd. The animals were fed under a specific pathogen-free environment in the Shanghai Xinhua Hospital animal laboratory. All animal experiments were conducted under the rules approved by the Shanghai Xinhua Hospital Ethics Committee.

### Platelet depletion

In order to investigate the role of platelets in NET formation and ALI development, platelets were depleted in WT C57BL/6 mice by injecting busulfan i.p. or anti-platelet antibody i.v. as previously described [23, 24], resulting in a 40% or 60% reduction of platelets, respectively, without affecting leukocytes.

### Mouse model of cecal ligation and puncture (CLP) and in vivo exosome administration

The mouse CLP model was prepared as previously described [25]. In total, 40 mice were used in sham group or in CLP group. At 24 h after surgery, WT male C57BL/6 mice were euthanized with a phenobarbital overdose (100 mg/kg body weight), after which bronchoalveolar lavage fluid (BALF) and citrate-anticoagulated whole blood were collected at a 1:7 ratio. In order to visualize morphological changes and NET formation in sepsis-induced ALI, lung tissue was harvested and fixed in 4% paraformaldehyde 24 h for H&E and immunofluorescence staining.

To explore exosome function in vivo, mice were treated with exosomes isolated from the plasma of sham or CLP mice (300 μg/mouse) through i.p. injection using 31-gauge insulin syringes. After 24 h, BALF, venous blood, and lung tissue were harvested as described previously.

### NET quantification assay

To quantify NETs in the cell culture supernatant, plasma, and mouse BALF, we used the PicoGreen dsDNA Quantification Kit (Invitrogen, Carlsbad, CA, USA) and a capture ELISA based on myeloperoxidase (MPO) associated with DNA [6]. For ELISA analysis of NET concentration, 1 μg/mL anti-MPO mAb was used as a capture antibody with Cell Death Detection ELISA (Roche, Indianapolis, IN, USA) according to the instructions.

### Sequencing of miRNA and data analysis

Exosomes were isolated from the supernatant of platelets stimulated with PBS (PBS-Exo) or LPS (LPS-Exo) ex vivo. Total RNA was extracted from exosomes using the miRNeasy Serum/Plasma Kit (Qiagen, Valencia, CA, USA). The final ligation PCR products were sequenced using the BGISEQ-500 platform (BGI Group, Shenzhen, China).

After acquiring the raw data, the differently expressed miRNAs were calculated using the *t* test. Those with ≥ 1.2-fold change and a *P* value < 0.05 were regarded as significantly different. A heat map was generated using the R 3.5.3 software. Pathway analysis was conducted using the reactome pathway database. The 20 most enriched pathways are listed and were used to reveal the most associated pathways.

### Statistics

The normal distribution of the data was tested using the Shapiro-Wilk test. For normal distribution data, data are presented as means ± SEM. Comparisons between 2 groups under identical conditions were performed by the 2-tailed Student’s *t* test. Multiple group comparisons were performed by one-way ANOVA followed by calculating the least significant difference to compare means. For data are not normally distributed, data are presented as median (25th–75th percentile). Comparisons between 2 groups were performed by Mann-Whitney *U* test. A value of *P* < 0.05 was considered statistically significant.

Sample size was determined by PASS 11 software (NCSS, LLC, Kaysville, UT, USA). The MPO-DNA complexes in plasma of three septic shock patients or healthy volunteers were taken in the preliminary test. The MPO-DNA complexes in plasma of septic shock patients and healthy volunteers were 0.23 ± 0.11 and 0.09 ± 0.04, respectively. Based on the difference between groups and assuming a two-sided type I error rate of 0.05 and a power of 0.80, 6 patients in each group were required to reveal a statistically significant difference. Besides, the power analysis was also performed to determine the number of animals used in this study to reach statistical significance. The MPO-DNA complexes in plasma of three sham or CLP mice were taken in the preliminary test. Three mice in each group were required to reveal a statistically significant difference.

## Results

### The elevation in NET formation is associated with mortality and severity during septic shock

**Fig. 1:**
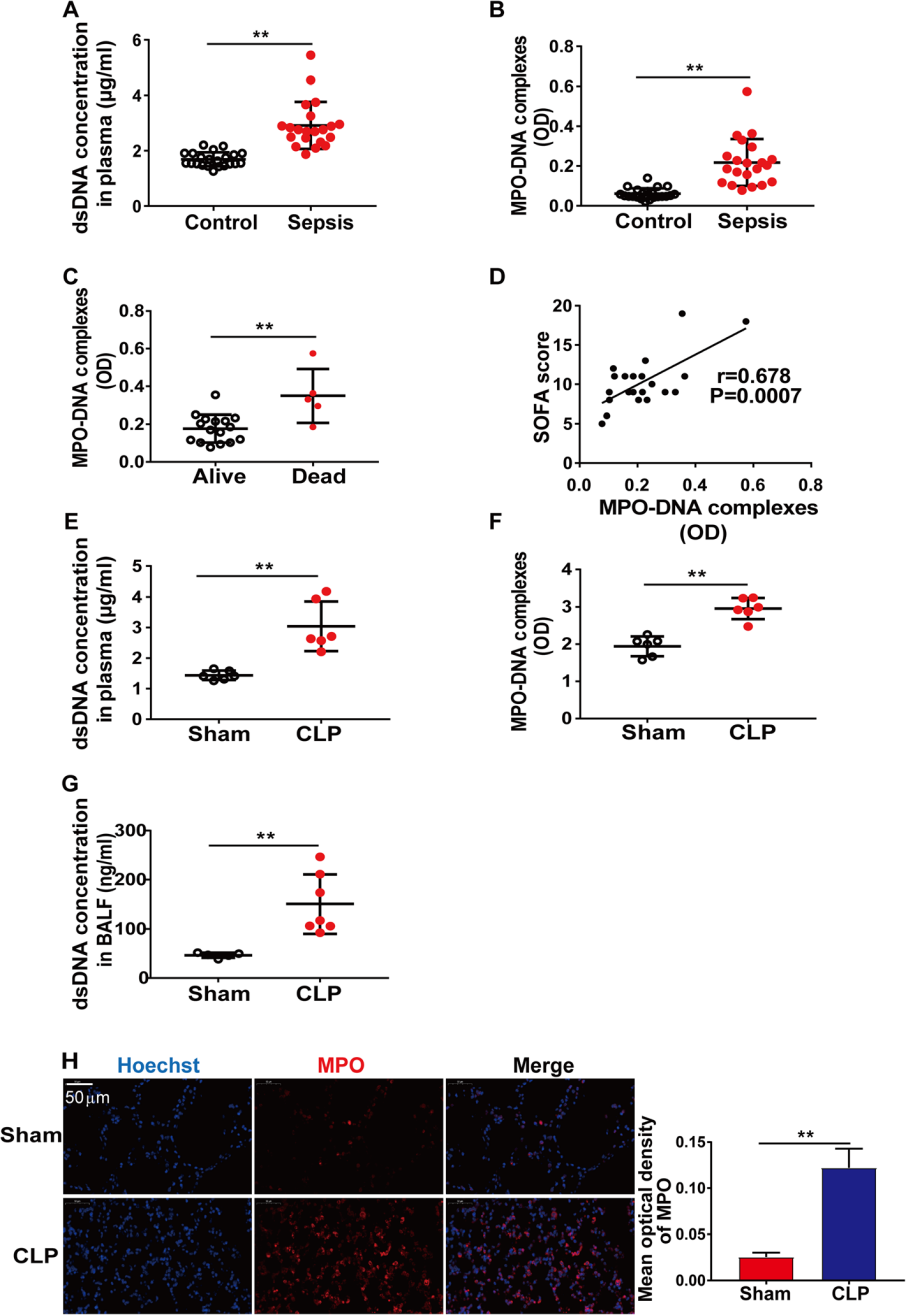
The elevation of NET formation is associated with mortality and severity during septic shock. **a**, **b** Quantification of dsDNA and circulating NET structures in the plasma of healthy controls (*n* = 22) and septic shock patients (*n* = 21) using PicoGreen fluorescent dye and MPO-DNA-ELISA, respectively. **c**, **d** Associations of MPO-DNA complexes with septic shock mortality and SOFA score. **e**–**h** Mice were subjected to sham or CLP for 24 h. **e**, **f** Quantification of dsDNA and circulating NET structures in the plasma of sham (*n* = 6) and CLP mice (*n* = 6). **g** Quantification of dsDNA in the BALF of sham (*n* = 5) and CLP mice (*n* = 7) using PicoGreen fluorescent dye. **h** Representative images of direct immunofluorescence staining of DNA (blue) and MPO (red) in the lung sections of sham and CLP mice. Scale bar, 50 μm. Graphs represent means ± SEM; **P < 0.01 compared within two groups

In this study, dsDNA and soluble NET components (MPO-DNA complexes) in human plasma were quantified by PicoGreen dsDNA quantification and ELISA, respectively. As shown in Fig. 1a and b, the amounts of plasma dsDNA and MPO-DNA complexes were significantly higher in septic shock patients than those in healthy controls. Greater NET formation was associated with poor prognosis (Fig. 1c) and positively correlated with SOFA scores in septic shock patients (*r* = 0.678, *P* = 0.0007) (Fig. 1d). In the CLP model, NET formation was robustly induced in both plasma and BALF (Fig. 1e–g). The NET component MPO was also increased in the lung tissue of the CLP group (Fig. 1h).

### NET formation is largely dependent on platelets in sepsis

**Fig. 2:**
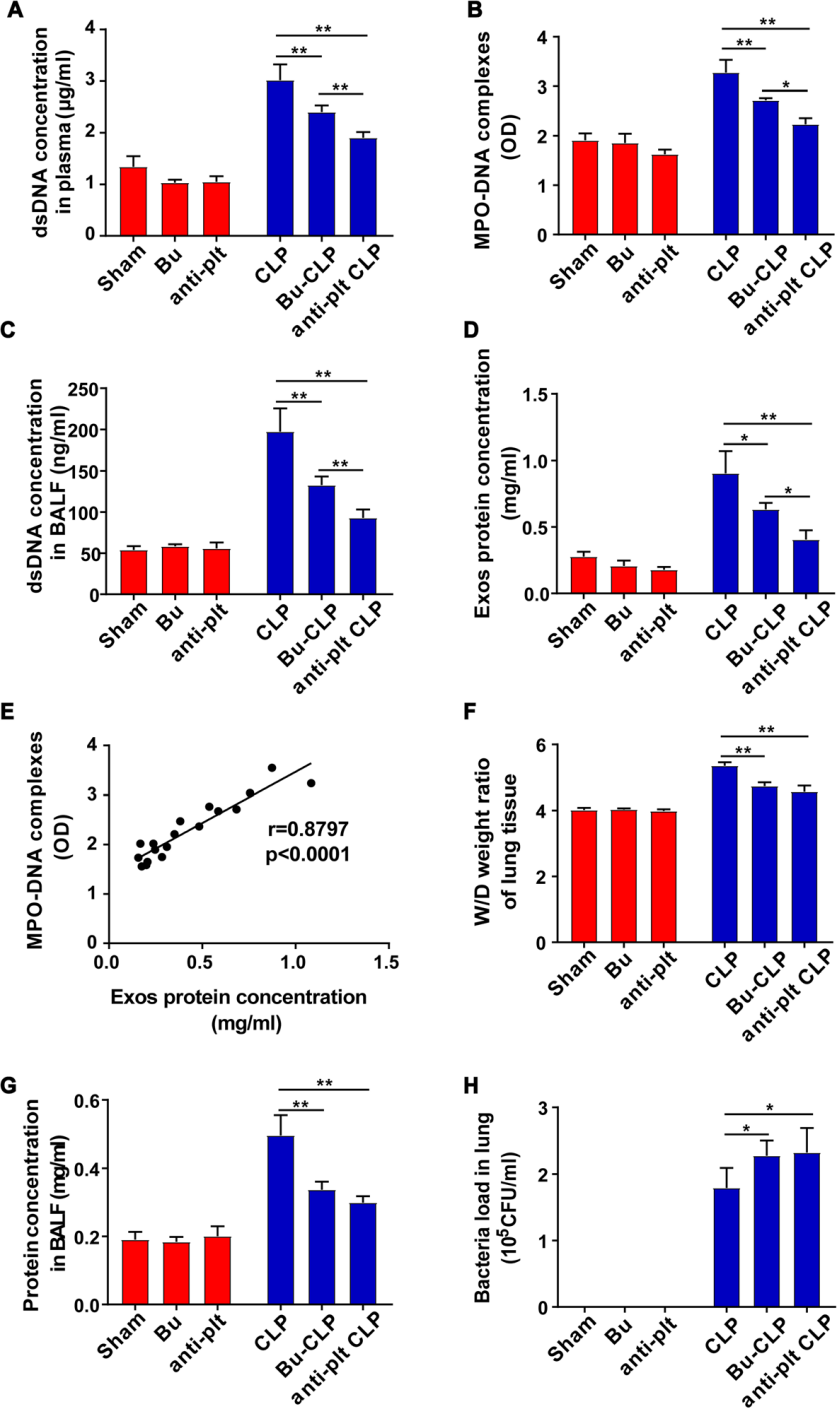
NET formation is largely dependent on platelets in sepsis. WT mice were treated with busulfan (25 mg/kg, i.p.) or anti-platelet Ab (50 μL/mouse, i.v.) prior to CLP. **a**, **b** Quantification of dsDNA and circulating NET structures in the plasma of mice using PicoGreen fluorescent dye and MPO-DNA-ELISA, respectively. **c** Quantification of dsDNA in BALF using PicoGreen fluorescent dye. **d** Exosomes were isolated from equal plasma volumes, and the protein concentration was determined using a BCA protein assay kit. **e** Plasma NETs and exosomal protein concentration are positively correlated (*r* = Pearson’s correlation coefficient). **f** At 24 h after sham or CLP, the wet/dry ratio of lung tissue was measured. **g** The total protein concentration in BALF was quantified using BCA. **h** Lung tissues were harvested at 24 h after CLP. Supernatant was made after homogenation and centrifugation. Equal amount of supernatant was spread on agar plates for colony formation. The number of bacterial colonies was assessed. Graphs represent means ± SEM, *n* ≥ 3; **P < 0.01 compared within two groups

Platelets have been implicated in promoting NET formation; therefore, to investigate their role in sepsis-induced NET formation, platelets were depleted by vehicle (polyethylene glycol 400) or busulfan and the platelet counts were measured by flow cytometry staining with FITC anti-mouse CD41 antibody (96.4%, 94.6–96.95% vs 55.8%, 53.95–57.05%). Platelets were also depleted by mouse IgG or anti-platelet Ab, which reduced blood platelet counts from 89.7% (89–89.95%) to 23.8% (20.7–26.45%). Platelet depletion by either busulfan or anti-platelet antibody markedly but not completely decreased the concentration of NET components in mouse plasma and BALF and reduced the exosome quantity in mouse plasma at 24 h following CLP (Fig. 2a–d). The results also showed that the plasma NET concentration was positively correlated with plasma exosome levels (Fig. 2e, *r* = 0.8797, *P* < 0.0001). Furthermore, the lung wet/dry (W/D) ratio and BALF protein concentration, which both reflect lung injury, were decreased in CLP mice following platelet depletion, while the lung injury was not completely alleviated by platelet depletion prior to CLP (Fig. 2f, g). However, the bacterial load in the lungs was increased after platelet depletion (Fig. 2h).

### Characterization of exosomes isolated from the plasma of septic shock patients

**Fig. 3:**
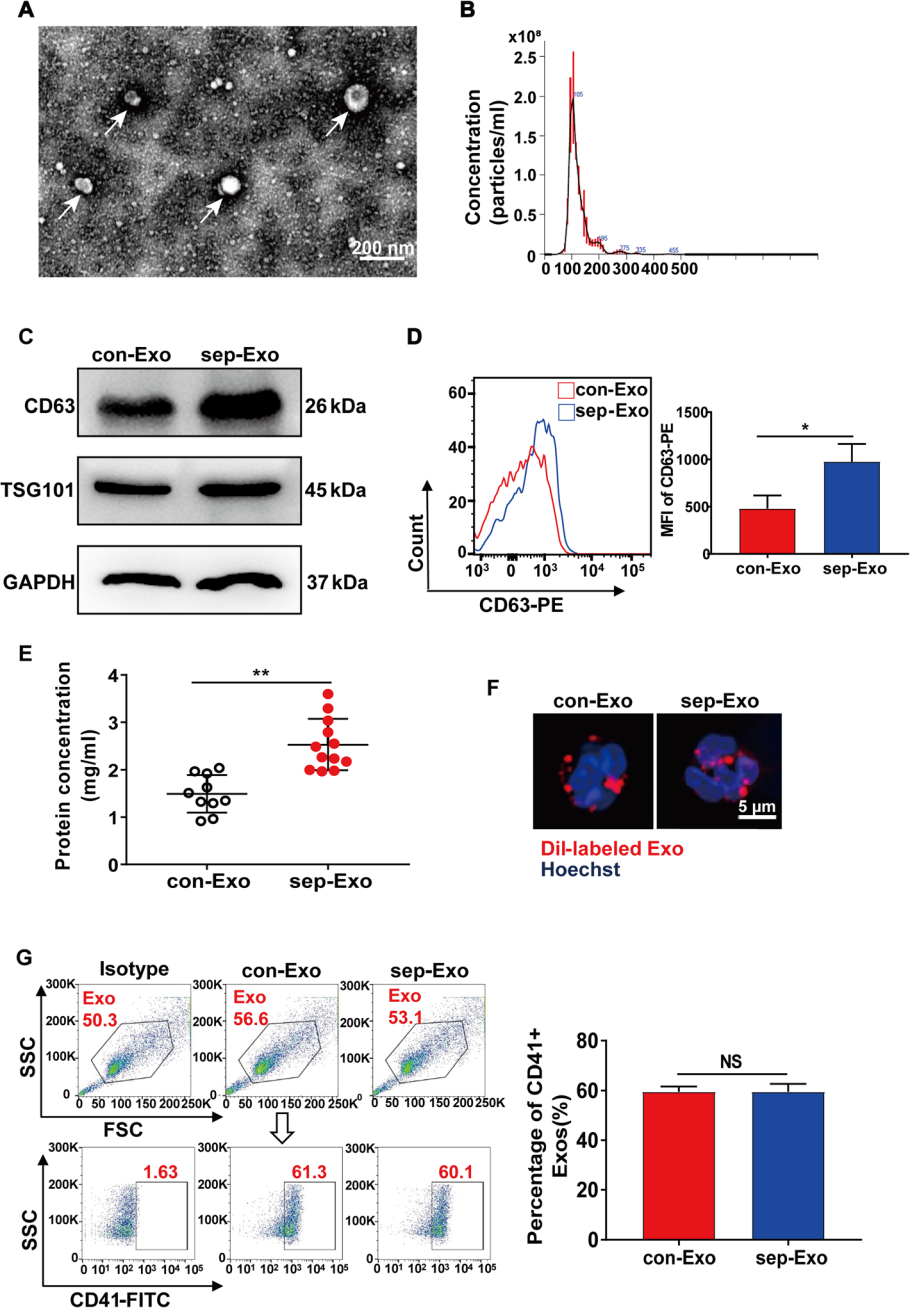
Characterization of exosomes isolated from the plasma of septic shock patients. **a** Electron micrograph of exosomes (indicated by white arrows) derived from the plasma of septic shock patients. Scale bar, 200 nm. **b** Plasma exosome size distribution was measured by NanoSight tracking analysis. **c** CD63, TSG101, and GAPDH protein expression in exosomes were quantified by western blot loaded with equal amounts of exosome protein (40 μg). **d** Exosomes were detected by CD63 expression using flow cytometry. MFI, mean fluorescence intensity. **e** Protein quantification of exosomes derived from 800 μL plasma from healthy controls (*n* = 10) and septic shock patients (*n* = 12) by BCA. **f** Immunofluorescence images showing PMNs incubated with Dil-labeled exosomes (red) for 2 h. Nuclei were counterstained with Hoechst (blue). Scale bar, 5 μm. **g** Platelet-derived exosomes were identified by CD41-FITC staining and detected by flow cytometry. The proportion of CD41-positive exosomes was calculated (*n* = 3). SSC, side scatter. Graphs represent means ± SEM; **P < 0.01 compared within two groups

Exosomes isolated from the plasma of human septic shock patients were first characterized morphologically using transmission electron microscopy. As shown in Fig. 3a, the isolated microvesicles displayed a round, cup-shaped morphology and were approximately 100 nm in diameter. Further, nanosight analysis determined that the particle size distribution of the purified exosomes was about 100 nm (Fig. 3b), and western blot showed high expression of exosome-specific markers CD63 and TSG101 in samples from both septic shock patients and healthy controls (Fig. 3c). Flow cytometry further showed that CD63 expression was higher in the exosomes from septic shock patients (sep-Exo) than in those from healthy controls (con-Exo) (Fig. 3d). Figure 3e showed that the protein concentration in sep-Exo was higher than that in con-Exo when isolated from equal plasma volumes (800 μL). After incubating PMNs in vitro with Dil-labeled exosomes for 2 h, we observed PMN internalization of exosomes (Fig. 3f). Besides, the internalization of exosomes in 9 h was not significantly higher than that in 3 h (Additional file 1: Figure S1). To determine the proportion of platelet-derived exosomes in the total plasma exosome quantity, we further identified exosomes by detecting CD41 expression, which is a platelet marker. We found that platelets accounted for ~ 60% in both sep-Exo and con-Exo; however, no differences were observed in platelet origin (59.57 ± 1.19 vs 59.57 ± 1.81, *P* > 0.9999) (Fig. 3g).

### NET formation was induced by platelet-derived exosomes during septic shock

**Fig. 4:**
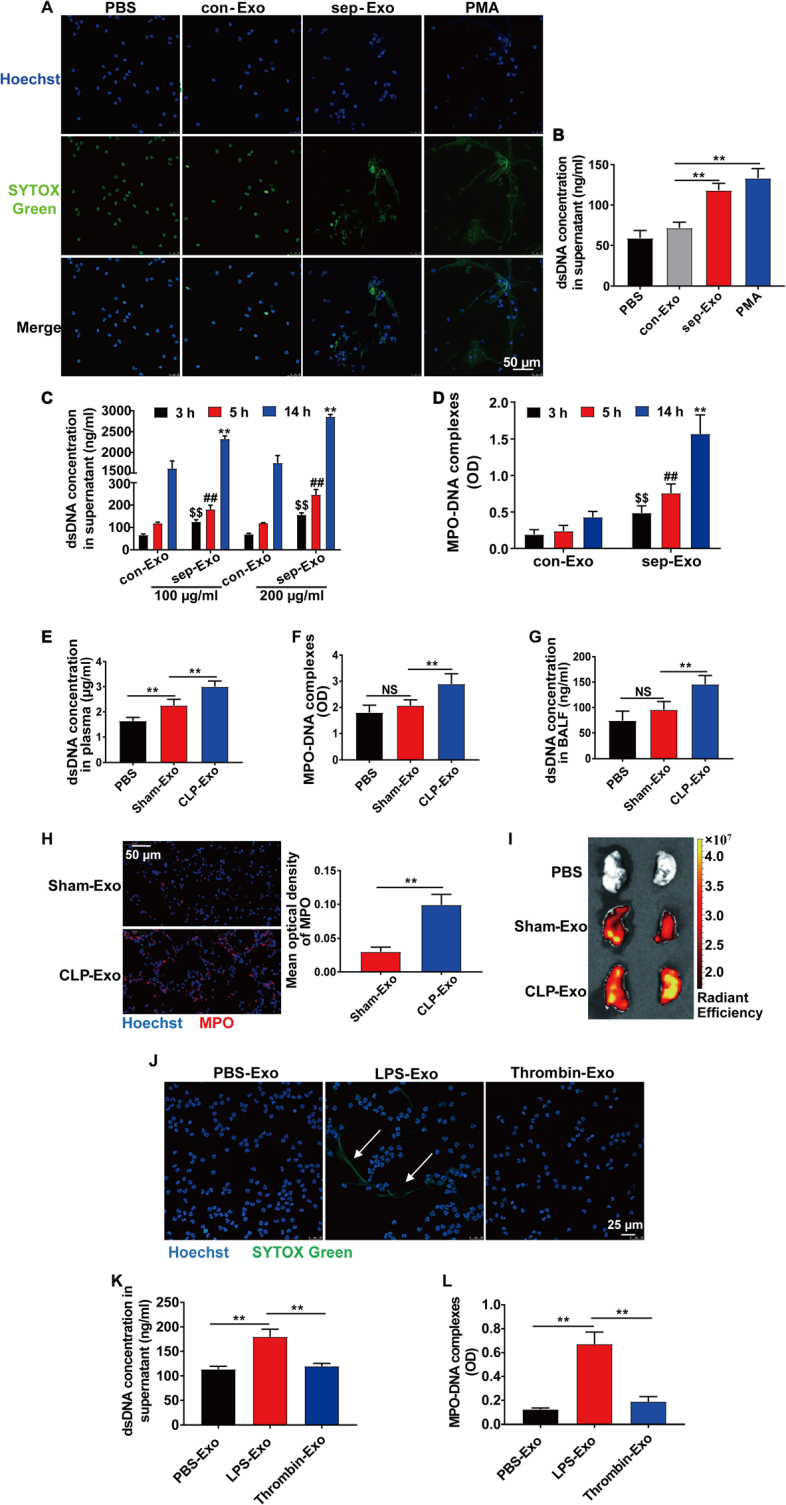
NET formation was induced by platelet-derived exosomes during septic shock. Exosomes were isolated from the plasma of healthy controls and septic shock patients. **a** PMNs isolated from the peripheral blood of healthy volunteers were cocultured with PBS or 100 μg/mL exosomes or 50 nM PMA for 3 h, and NET formation was visualized by confocal microscopy using the following fluorescent dyes: Hoechst 33342 (blue) and SYTOX Green (green). **b** Quantification of dsDNA in the supernatant of cultured PMNs using PicoGreen fluorescent dye. **c** PMNs were cocultured with various exosome concentrations for different periods, and the supernatant dsDNA concentration was measured using PicoGreen fluorescent dye. **d** PMNs were cocultured with exosomes (100 μg/mL) for different periods, and the MPO-DNA complexes were measured using ELISA. **e**–**h** Mice were injected with PBS or exosomes (300 μg/mouse, i.p.) isolated from the plasma of sham or CLP mice for 24 h. **e**, **f** Quantification of dsDNA and circulating NET structures in the plasma of mice using PicoGreen fluorescent dye and MPO-DNA-ELISA, respectively. **g** Quantification of dsDNA in BALF using PicoGreen fluorescent dye. **h** Representative images of direct immunofluorescence staining of DNA (blue) and MPO (red) in lung sections after exosome injection. **i** Ex vivo fluorescence signals in the lungs of mice injected i.p. with Dil-labeled exosomes from equal plasma volumes (100 μL). **j**–**l** Platelets were stimulated with PBS or 1 μg/mL LPS or 0.1 U/mL thrombin for 3 h. Exosomes were isolated from the supernatant of stimulated platelets and cocultured with PMNs for 5 h. **j** NET formation was visualized by confocal microscopy. **k**, **l** Quantification of dsDNA and circulating NET structures in the supernatant of cultured PMNs using PicoGreen fluorescent dye and MPO-DNA-ELISA, respectively. All results are representative of 3 independent experiments. Graphs represent means ± SEM; **P < 0.01 compared within two groups

To test the effect of platelet-derived exosomes on NET formation during septic shock, we cocultured PMNs isolated from the peripheral blood of healthy volunteers with con-Exo or sep-Exo for 3 h. Confocal microscopy and fluorescence intensity assay demonstrated that sep-Exo was able to induce NET formation (Fig. 4a, b), and this ability was time- and dose-dependent (Fig. 4c, d). For in vivo animal study, C57bl/6 WT mice were injected with sham-Exo or CLP-Exo (300 μg/mouse, i.p.) for 24 h. CLP-Exo increased the NET concentration in plasma and BALF (Fig. 4e–g) and upregulated the expression of MPO in lung tissue (Fig. 4h). When injected with Dil-labeled exosomes from equal plasma volumes, CLP-Exo accumulated more in the lung than Sham-Exo (Fig. 4i).

In addition, to further confirm that platelet-derived exosomes induce NET formation, human platelets were stimulated ex vivo with PBS, LPS, or thrombin. Exosomes isolated from the supernatant of PBS-stimulated platelets were characterized morphologically using transmission electron microscopy (Additional file 1: Figure S2A). The protein concentrations of LPS-Exo and thrombin-Exo were higher than those of PBS-Exo (Additional file 1: Figure S2B). Exosomes were cocultured with PMNs for 5 h, and NET formation was measured by confocal microscopy, fluorescence intensity assay, and ELISA. The data showed that exosomes from LPS-stimulated platelets (LPS-Exo) induced NET formation (Fig. 4j–l). In addition, we collected supernatant of PBS/LPS/thrombin-stimulated platelets and depleted exosomes by ultracentrifugation, then cultured freshly isolated human PMNs in exosome-free supernatant and found that the exosome-free supernatant of LPS/thrombin-stimulated platelets significantly increased NET concentration (Additional file 1: Figure S2C, S2D).

Collectively, the results indicate that platelet-derived exosomes induce NET formation during septic shock.

### Platelet-derived exosomes induce NET formation through modulating the Akt/mTOR-related autophagy pathway

**Fig. 5:**
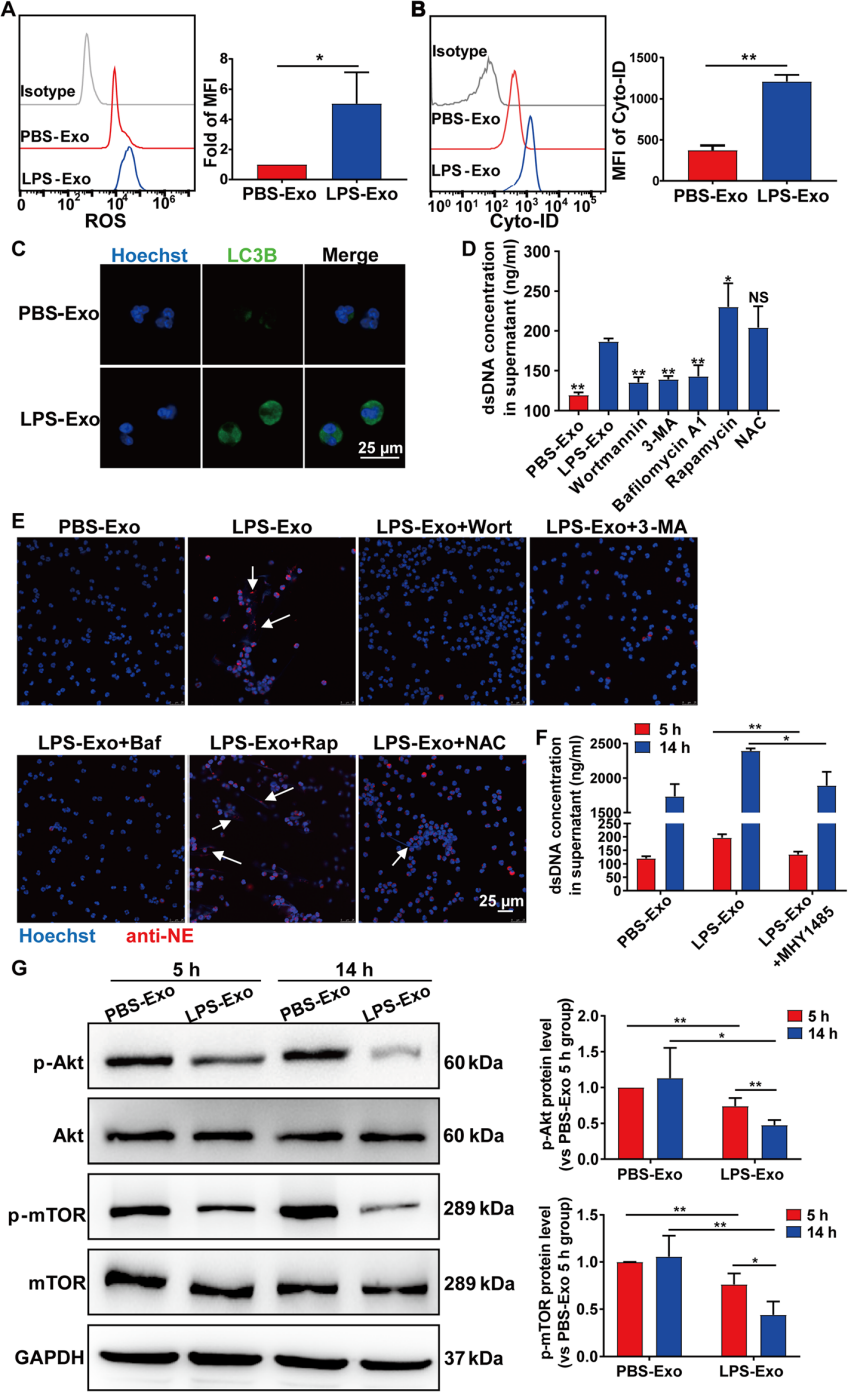
Platelet-derived exosomes induce NET formation through modulating the Akt/mTOR-related autophagy pathway. PMNs were cocultured with exosomes isolated from the supernatant of platelets stimulated with PBS (PBS-Exo) or LPS (LPS-Exo) for 5 h. **a** ROS expression in PMNs was measured by flow cytometry with CM-H2DCFDA staining. **b** PMNs autophagy was assessed by staining with Cyto-ID autophagy tracer. **c** Immunofluorescence images showing LC3B expression (green) in PMNs. Nuclei were counterstained with Hoechst (blue). **d**, **e** PMNs were cocultured with PBS-Exo or LPS-Exo with or without wortmannin (150 nM), 3-MA (5 mM), bafilomycin A1 (1 μM), rapamycin (100 nM), and NAC (50 μg/mL). **d** The supernatant dsDNA concentration was quantified by PicoGreen. **e** NET formation was visualized by confocal microscopy with the following fluorescent dyes: Hoechst 33342 (blue) and anti-neutrophil elastase (anti-NE; red). **f** PMNs were cocultured with PBS-Exo or LPS-Exo in the presence or absence of MHY1485 (2 μM). The supernatant dsDNA concentration was quantified by PicoGreen. **g** Western blot of p-Akt, Akt, p-mTOR, and mTOR in PMNs after coculturing with exosomes. All results are representative of 3 independent experiments. Graphs represent means ± SEM; *P < 0.05, **P < 0.01 compared within two groups

The mechanisms that induce NETs are not fully understood. Previous studies have revealed that NET formation involves reactive oxygen species (ROS) generation and autophagy, which are mainly dependent on stimuli [9, 26]. To test which process is involved in platelet-derived exosome-induced NET formation, we examined ROS production and autophagy activation in PMNs after coculturing with exosomes. As shown in Fig. 5a and Additional file 1: Figure S3A, platelet-derived exosomes increased ROS production in PMNs after coculturing for 5 h. Autophagic activity was examined in PMNs isolated from healthy controls and septic shock patients by evaluating the lipidation of LC3B, Atg5, and p62 expression (Additional file 1: Figure S3B), as well as by flow cytometry to measure the intracellular accumulation of Cyto-ID, a dye that selectively labels autophagic vacuoles (Additional file 1: Figure S3C). The data showed that septic shock patients had a relatively higher autophagy level in PMNs. Furthermore, autophagic activity was also upregulated in PMNs after coculturing with sep-Exo or LPS-Exo for 5 h in vitro, as assessed by confocal microscopy and flow cytometry (Fig. 5b, c, Additional file 1: Figure S3D). Besides, the exosome-free supernatant of LPS-stimulated platelets increased PMNs autophagic activity as compared to the exosome-free supernatant of PBS-stimulated platelets (Additional file 1: Figure S2E).

Collectively, these results indicate that platelet-derived exosomes increase ROS production and autophagy level in PMNs during septic shock. Next, autophagy inhibitors (wortmannin, 3-MA, and bafilomycin A1), an autophagy inducer (rapamycin), or an ROS inhibitor (NAC) were added to PMNs prior to coculturing with exosomes. As assessed by fluorescence intensity assay and confocal microscopy, NET formation was inhibited by autophagy inhibitors, but not by the ROS inhibitor, and was further induced by rapamycin after coculturing with LPS-Exo (Fig. 5d, e). The addition of wortmannin, 3-MA, or bafilomycin A1 to LPS-Exo increased the percentage of apoptotic cells, suggesting that autophagic activity plays a regulatory role (Additional file 1: Figure S3E).

Mammalian target of rapamycin (mTOR) is a dominant regulator of autophagy induction that inhibits autophagosome formation, and mTOR regulation by serine/threonine kinase Akt is well established. It is generally assumed that alterations in Akt signaling would have a great impact on autophagy [27]. As shown in Fig. 5f, MHY1485-enhanced mTOR phosphorylation suppressed LPS-Exo induced NET formation. Moreover, we found that the phosphorylated mTOR (p-mTOR) and p-Akt levels were reduced after coculturing with LPS-Exo, while the p-mTOR and p-Akt levels were even lower after exosome treatment for 14 h than those after 5 h (Fig. 5g).

Altogether, these results suggest that platelet-derived exosomes increase autophagic activity in PMNs through modulating the Akt/mTOR pathway and eventually lead to greater NET formation during septic shock.

### Platelet-derived exosomes induce NET formation via HMGB1

**Fig. 6:**
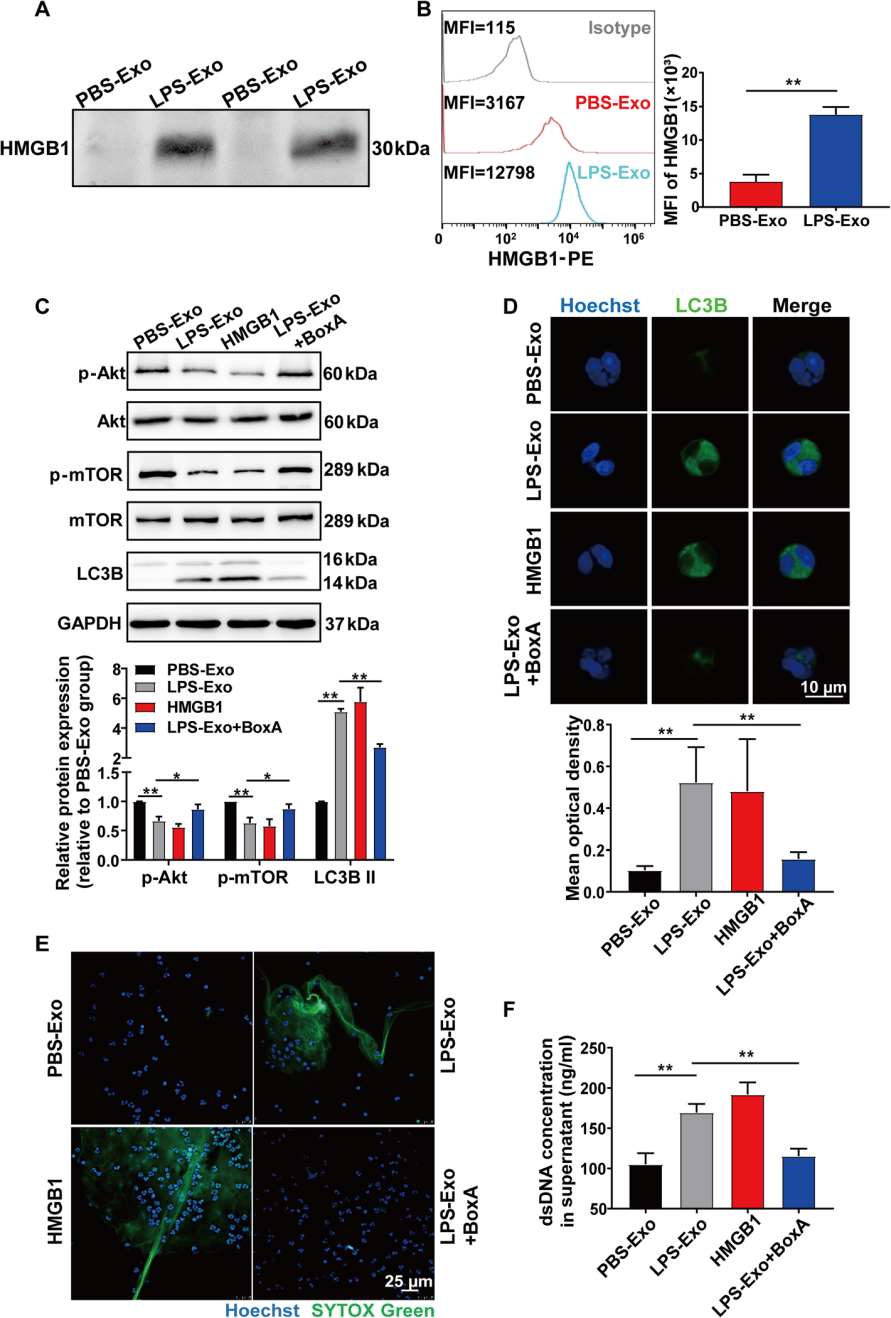
Platelet-derived exosomes induce NET formation via HMGB1. **a** HMGB1 expression in exosomes was determined by western blot loaded with equal amounts of exosomal protein (6 μg/μL, 40 μg). **b** Exosomes were detected by HMGB1 expression using flow cytometry. **c**–**f** PMNs were cocultured with PBS-Exo or LPS-Exo, stimulated with HMGB1 (1 μg/mL), or cocultured with LPS-Exo accompanied by BoxA (10 μg/mL) for 5 h. **c** Western blot of p-Akt, Akt, p-mTOR, mTOR, and LC3B in PMNs. **d** Immunofluorescence images show LC3B expression (green) in PMNs. Nuclei were counterstained with Hoechst (blue). **e** NET formation was visualized by confocal microscopy with the following fluorescent dyes: Hoechst 33342 (blue) and SYTOX Green (green). **f** Quantification of dsDNA in the supernatant of cultured PMNs using PicoGreen fluorescent dye. All results are representative of 3 independent experiments. Graphs represent means ± SEM; *P < 0.05, **P < 0.01 compared within two groups

Previous studies have revealed that platelet HMGB1 activates PMNs and induces NET generation [14, 18]. HMGB1 is a known inducer of autophagy. Therefore, we hypothesized that platelet-derived exosomes induce NET formation via HMGB1. First, we examined the presence of HMGB1 in exosomes and found a significantly higher HMGB1 expression level in LPS-Exo than in PBS-Exo, as assessed by western blot and flow cytometry (Fig. 6a, b). Besides, HMGB1 concentration in exosome-free supernatant of LPS-stimulated platelets was significantly higher than that in exosome-free supernatant of PBS-stimulated platelets, as assessed by ELISA (Additional file 1: Figure S2F).

The expression levels of p-Akt and p-mTOR were increased and LC3B lipidation was inhibited in the presence of BoxA, an HMGB1 competitive antagonist (Fig. 6c). The same result was obtained by LC3B staining, as assessed by confocal microscopy (Fig. 6d). Further, NET formation was also abrogated by BoxA under LPS-Exo treatment (Fig. 6e, f). Taken together, these results indicate that platelet-derived exosomes induce NET formation via HMGB1.

### Exosomal miR-15b-5p and miR-378a-3p promote NET formation by targeting PDK1

**Fig. 7:**
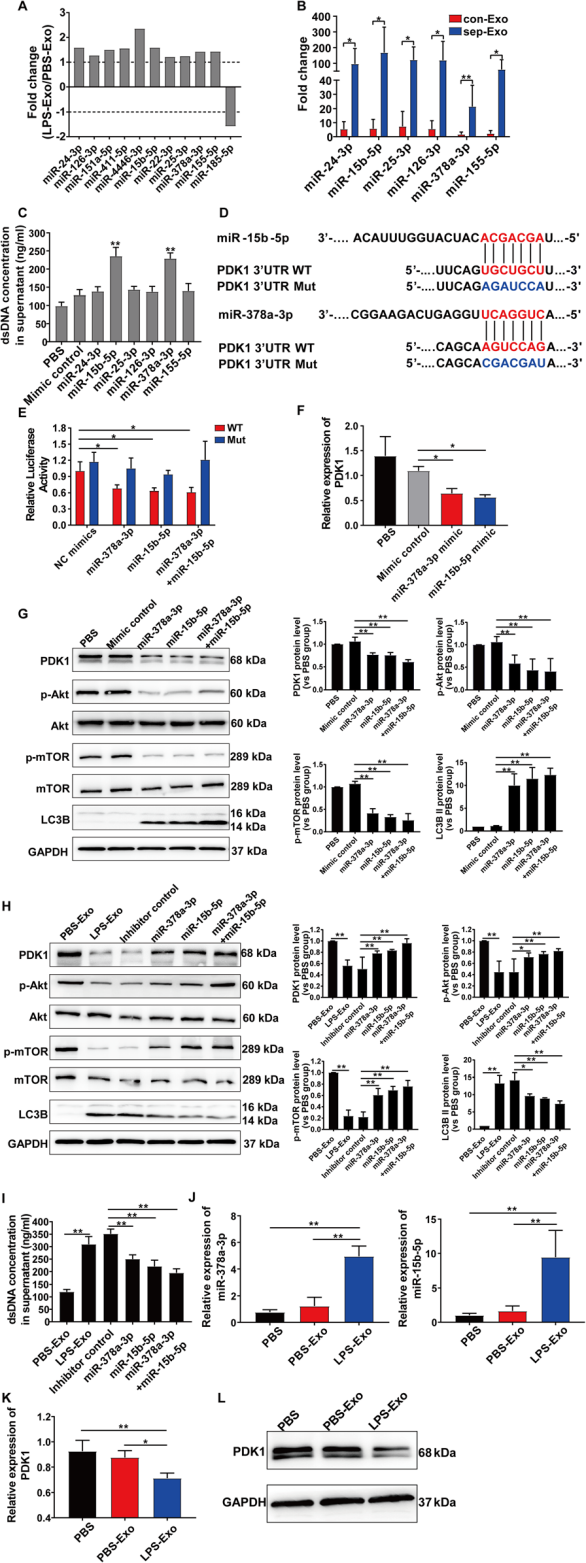
Exosomal miR-15b-5p and miR-378a-3p promote NET formation by targeting PDK1. **a** Differential expression of miRNAs between PBS-Exo and LPS-Exo is expressed as fold change, as determined by miRNA-seq. **b** qRT-PCR verified the miRNAs identified by miRNA-seq; results are shown as fold change (*n* = 6). **c** The overexpression of selected miRNAs (miR-24-3p, miR-15b-5p, miR-25-3p, miR-126-3p, miR-378a-3p, and miR-155-5p) was analyzed to assess their effect on NET formation. The supernatant dsDNA concentration was quantified by PicoGreen fluorescent dye. **d** Sequence alignment between miR-15b-5p, miR-378a-3p, and their putative binding sites (in red letters) in the PDK1 3′-UTR. Mutation of the miR-15b-5p and miR-378a-3p target sites (in blue letters) is shown below. **e** Luciferase reporter assay was performed to detect the relative luciferase activities of WT and Mut PDK1 reporters. The Renilla luciferase vector was used as an internal control. qRT-PCR analysis of relative PDK1 mRNA levels (**f**) and western blot (**g**) of PDK1, p-Akt, Akt, p-mTOR, mTOR, and LC3B in differentiated HL-60 cells transfected with miR-15b-5p or miR-378a-3p mimics as indicated. **h**, **i** Prior to coculturing with LPS-Exo for 24 h, differentiated HL-60 cells were transfected with control, miR-15b-5p or miR-378a-3p inhibitors, or both for 24 h. **h** The expression levels of PDK1, p-Akt, Akt, p-mTOR, mTOR, and LC3B in differentiated HL-60 cells were measured by western blot. **i** Quantification of dsDNA in the supernatant of differentiated HL-60 cells using PicoGreen fluorescent dye. **j**–**l** PMNs were cocultured with PBS, PBS-Exo, or LPS-Exo for 14 h. Relative expression levels of miR-15b-5p, miR-378a-3p, and PDK1 were measured by qRT-PCR (**j**, **k**). Western blot of PDK1 in PMNs (**l**). Graphs represent means ± SEM, *n* = 3; *P < 0.05, **P < 0.01 compared within two groups

Previous studies have suggested that exosomes from septic shock patients convey miRNAs related to pathogenic pathways, which may represent a novel mechanism for intercellular communication during sepsis [17]. In addition, it takes time for miRNAs to form RNA-induced silencing complex to degrade mRNA or prevent mRNA translation [28]. Therefore, we tested the role of miRNAs in platelet-induced NET formation in a later phase. As shown in Additional file 1: Figure S4A, we screened PBS-Exo and LPS-Exo for miRNAs and detected 84 differentially expressed miRNAs. The sequencing data have been deposited in the NCBI Sequence Read Archive (SRA) database under the accession number PRJNA590319. Enrichment pathway analysis was also performed to identify the most enriched pathways for these 84 miRNAs, and the data showed that platelet activation and negative regulation of the phosphatidylinositol 3-kinase (PI3K)/Akt network were within the 20 most enriched pathways (Additional file 1: Figure S4B). Next, we identified the differentially expressed miRNAs that target the Akt/mTOR pathway; only experimentally validated targets were selected, which are indicated by arrows in Additional file 1: Figure S4A. The relative expression of the 11 miRNAs identified by miRNA-seq is shown in Fig. 7a. Follow-up qRT-PCR verification showed that miR-24-3p, miR-15b-5p, miR-25-3p, miR-126-3p, miR-378a-3p, and miR-155-5p were significantly higher in sep-Exo than in con-Exo (Fig. 7b). Functional analysis was conducted by transfecting differentiated HL-60 cells with miRNA mimics or mimic control, and the data showed that only miR-15b-5p and miR-378a-3p increased the dsDNA concentration in the supernatant (Fig. 7c). Collectively, these results suggest that platelet-derived exosomes induce NET formation via miR-15b-5p and miR-378a-3p.

Next, we searched the literature for a possible target of miR-15b-5p and miR-378a-3p and found that phosphoinositide-dependent protein kinase 1 (PDK1), which activates Akt by phosphorylation at Thr-308, contains a highly conserved putative miR-378 binding site in its 3′-UTR. We thus used TargetScan to predict the target genes and identified PDK1 as a potential target of miR-15b-5p and miR-378a-3p (Fig. 7d). To validate this bioinformatic prediction, luciferase assay was conducted. The WT or Mut 3′-UTR of the PDK1 sequence containing the predicted miR-15b-5p or miR-378a-3p binding sites was cloned into the plasmid vector, which was then transfected into HEK293 cells. Then, the miR-15b-5p or miR-378a-3p mimic or control was transfected into HEK293 cells, and the luciferase activity was tested. Luciferase activity was strikingly inhibited by miR-15b-5p and miR-378a-3p mimic in the PDK1 3′-UTR WT group, but not in the PDK1 3′-UTR Mut group (Fig. 7e). Moreover, miR-15b-5p or miR-378a-3p mimic decreased the relative expression of PDK1 mRNA (Fig. 7f); western blot demonstrated the same result, as the PDK1 protein level decreased in differentiated HL-60 cells after transfection with miR-15b-5p or miR-378a-3p mimic (Fig. 7g). The above results confirmed that PDK1 was the direct target of miR-15b-5p and miR-378a-3p.

Next, to test whether miR-15b-5p and miR-378a-3p could modulate the Akt/mTOR pathway by targeting PDK1, we transfected differentiated HL-60 cells with miRNA mimics or inhibitors prior to coculturing with or without LPS-Exo. As shown in Fig. 7g, miR-15b-5p and miR-378a-3p mimics downregulated p-Akt and p-mTOR levels and upregulated the LC3B II protein level. Transfection of miR-15b-5p or miR-378a-3p inhibitors abrogated the ability of LPS-Exo to decrease PDK1, p-Akt, and p-mTOR and increase LC3B lipidation (Fig. 7h). In addition, NET formation induced by LPS-Exo was inhibited by miR-15b-5p or miR-378a-3p inhibitors (Fig. 7i).

To test whether the miR-15b-5p/miR-378a-3p-PDK1 pathway is truly involved in exosome regulation, we investigated the miR-15b-5p, miR-378a-3p, and PDK1 levels in PBS-Exo- or LPS-Exo-treated PMNs. After 14 h, the expression levels of miR-15b-5p and miR-378a-3p were significantly higher in the LPS-Exo group (Fig. 7j). PDK1 mRNA and protein levels were reduced by LPS-Exo treatment (Fig. 7k, l).

Taken together, these results indicate that platelet-derived exosomal miR-15b-5p and miR-378a-3p inhibited Akt/mTOR pathway activity by targeting PDK1 in PMNs, thus promoting NET formation.

### IKK controls platelet-derived exosome secretion during septic shock

**Fig. 8:**
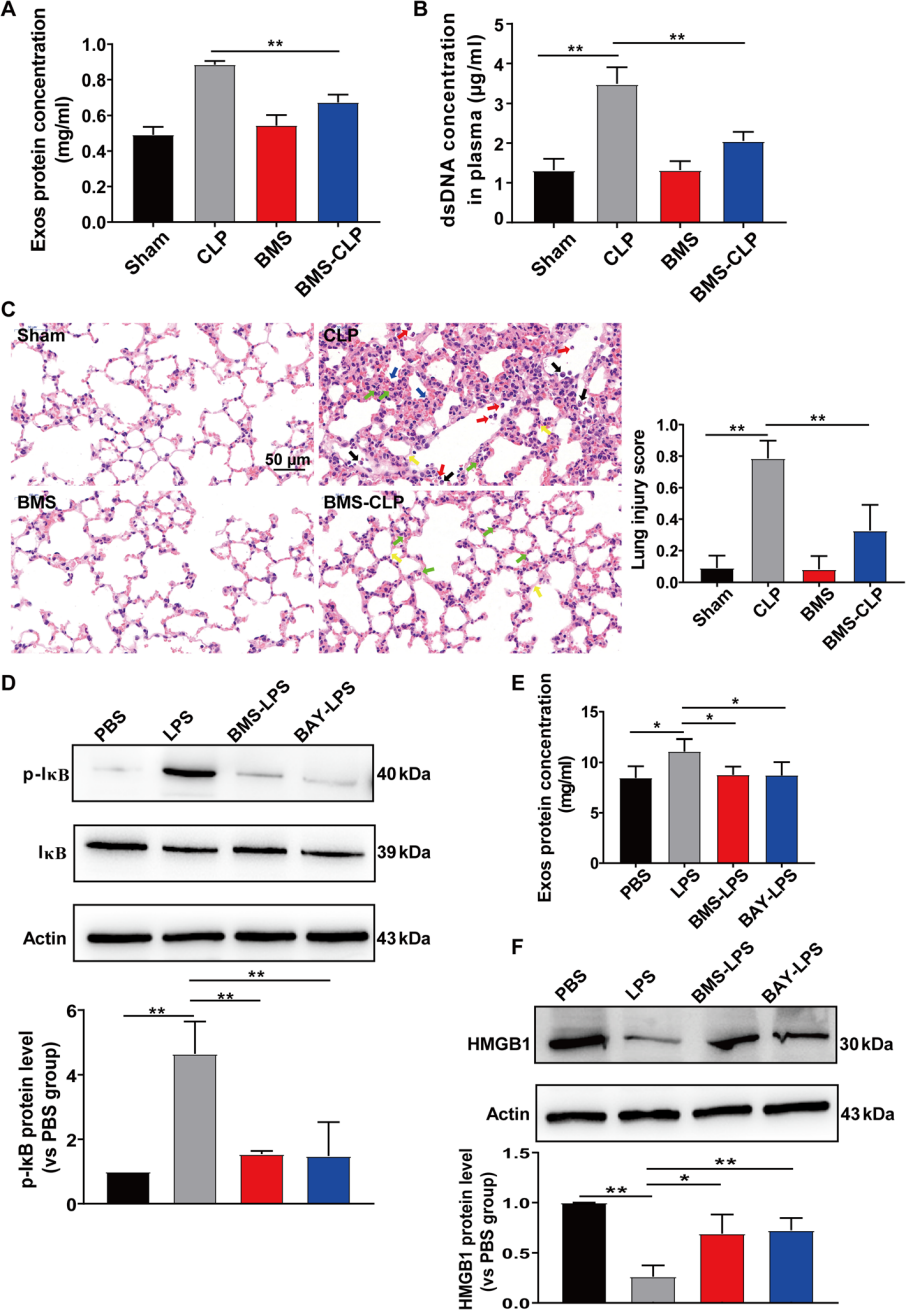
IKK controls platelet-derived exosome secretion during septic shock. **a**–**c** Mice were treated with either 3% Tween 80 (5 mL/kg, vehicle) or 10 mg/kg BMS-345541 in 3% Tween 80 by oral gavage. Two hours post-treatment, sham or CLP procedures were conducted. *n* = 5 mice per group. **a** Exosomes were isolated from equal plasma volumes (100 μL), and the protein concentration was determined using a BCA protein assay kit. **b** Quantification of dsDNA in the plasma of mice using PicoGreen fluorescent dye. **c** Lung histology was assessed with H&E staining (magnification × 400). The sham group showed normal lung tissue with thin alveolar walls and few alveolar macrophages. Red arrows indicate neutrophils in the alveolar space, green arrows indicate neutrophils in the interstitial space, black arrows indicate alveolar macrophages, yellow arrows indicate hyaline membranes, and blue arrows indicate thickening of the alveolar walls. Lung injury scores were assessed. Scale bar, 50 μm. **d**–**f** Platelets were pre-incubated with IKK inhibitors (10 μM BMS-345541 or 25 μM BAY 11–7082) for 5 min and stimulated with LPS (1 μg/mL) for 2 h. *n* = 3. **d** The expression levels of p-IκB and IκB in platelets were measured by western blot. **e** Exosomal protein levels in the supernatant of stimulated platelets were measured using BCA. **f** HMGB1 expression in platelets was measured by western blot. Graphs represent means ± SEM; *P < 0.05, **P < 0.01 compared within two groups

Next, we determined the mechanism that regulates platelet-derived exosome secretion during septic shock. Previous study suggests that SNAP-23 phosphorylation by IKK controls platelet secretion [29]. We administered IKK inhibitor (BMS-345541, 10 mg/kg) to mice 2 h prior to CLP and found that the plasma concentrations of exosomal protein and dsDNA were decreased (Fig. 8a, b). As shown in Fig. 8c, H&E staining demonstrated that IKK inhibitor alleviated lung injury following CLP. In addition, IKK inhibitors (BMS-345541 or BAY 11-7082) effectively inhibited IκB phosphorylation in LPS-stimulated platelets (Fig. 8d). Accordingly, exosome secretion in the supernatant of LPS-stimulated platelets was inhibited by BMS-345541 or BAY 11-7082 (Fig. 8e). The HMGB1 protein level in platelets after LPS stimulation was significantly decreased, and this effect was abrogated by IKK inhibitors (Fig. 8f). Collectively, these results suggest that platelet exosome secretion during septic shock was controlled by IKK.

## Discussion

In the current study, we demonstrate that NET components are significantly increased in the plasma of septic shock patients and correlate positively with disease severity and outcome. Platelet depletion in mice reduced the plasma exosome concentration, NET formation, and lung injury following sepsis. We also showed that exosomes isolated from the plasma of septic shock patients or from the supernatant of LPS-stimulated platelets induced NET formation. We further demonstrated that platelet-derived exosomes induce activation of the intra-neutrophil Akt/mTOR-related autophagy pathway and subsequent NET formation via exosomal HMGB1 and/or miR-15b-5p and miR-378a-3p. In addition, we revealed that IKK activity in platelets regulates exosome secretion during septic shock.

Septic shock remains one of the most challenging medical conditions, with increasing incidence in recent years. Accumulating experimental and clinical evidence indicates that overactive NET formation during sepsis can lead to the development of multiple organ dysfunction, highlighting the pathophysiological role of NETs in sepsis [30, 31]. Platelets have been shown to be potent activators of NET formation by direct platelet-neutrophil interactions via cell adhesion molecules or soluble mediators, such as HMGB1, platelet-derived chemokines, or thromboxane A2 [8]. Platelet depletion could inhibit NET formation and improve lung injury in multiple lung injury murine models [6, 23, 32]. In accordance with previous studies, we demonstrated that NETs are a hallmark of septic shock, and platelets promoted NET formation and lung injury during sepsis. Although platelets may serve as a target to alleviate lung injury, they also function as key elements in hemostasis and thrombosis. Current anti-platelet therapies invariably cause bleeding as an undesired adverse effect [33]. In addition, NETs have been proposed as an innate defense mechanism that is responsible for pathogen clearance, and impaired NET generation results in the dissemination of infections. As shown in our results, platelet depletion slightly increased the bacterial load in the lungs. Therefore, a critical balance of NETs is necessary to prevent lung injury and maintain microbial control [34].

This study shows that platelet-derived exosomes are major mediators that induce NET formation in septic shock. The results revealed that platelet depletion prior to CLP significantly decreased the plasma exosome concentration and reduced NET generation, and a positive relationship was found between platelet-derived exosomes and NETs. Previous studies have demonstrated that most exosomes accumulated in the blood of patients with sepsis are derived from platelets and correlate with organ dysfunction [11, 35, 36]. Our results suggested that sep-Exo or LPS-Exo from platelets promoted NET generation. Platelet TLR4 is involved in inducing NETs in mice and humans [24]; moreover, it plays prominent roles in sensing high circulatory LPS levels during sepsis and in neutrophil-mediated pathogen clearance [37]. However, the exact role of TLR4 in LPS-activated platelet exosome secretion needs to be further addressed.

The signaling pathways that mediate NET formation remain inadequately elucidated and have been found to vary in response to different stimuli [26, 38]. ROS generation by NADPH oxidase is an integral, but not essential, cellular process involved in NET formation [4]. The activation of NADPH and the MAPK-ERK pathways appears to be relevant, at least for NETs induced by PMA-activated neutrophils [39]. In addition to ROS generation, NET formation also requires autophagy [40]. Our previous study suggested that autophagy plays an important role in maintaining the function of NET formation in response to infection and in regulating neutrophil death [25]. Our present study showed that platelet-derived exosomes increased both the ROS level and autophagic activity in PMNs during septic shock, while pharmacological inhibition of ROS and autophagy demonstrated that only the autophagy pathway participated in platelet-derived exosome-induced NET generation. Our results were consistent with previous research that showed that NADPH inhibition did not interfere with NET formation induced by LPS-, Pam3CSK4-, or AA-stimulated platelets, suggesting that ROS are not NET mediators under these conditions [9].

One key regulator of autophagy is mTOR, a serine/threonine kinase that regulates cell growth, proliferation, and protein synthesis [27]. The PI3K/Akt/mTOR signaling pathway is a negative regulator of both autophagy and apoptosis. The inhibition of Akt/mTOR activity is known to play an essential role in initiating autophagy [41, 42]. In our study, platelet-derived exosomes promoted NET formation by inhibiting Akt/mTOR pathway activity during septic shock. Previous studies also demonstrated that HMGB1, either soluble or presented from activated platelets, induces autophagy in neutrophils, thus promoting NET generation [14, 18]. Therefore, we decided to address whether platelet-derived exosomes induced NET formation via HMGB1 during septic shock. Our results showed that the HMGB1 expression level was elevated during septic shock in platelet-derived exosomes, which recapitulated the effect of HMGB1 on the inhibition of Akt/mTOR activity and the induction of NET formation. BoxA abrogated all events elicited by platelet-derived exosomes.

Exosomally transferred miRNAs are emerging as novel regulators of cellular function. Evidence has been found in both immune cells and other cell types that transferred miRNAs repress target mRNAs in recipient cells. In the current study, we determined that exosomal miR-15b-5p and miR-378a-3p were involved in negatively regulating the activity of the Akt/mTOR pathway by repressing PDK1 expression. Recent study also demonstrated that miR-378 promotes autophagy initiation through the mTOR/unc-51-like autophagy activating kinase 1 pathway and sustains autophagy by targeting PDK1 [43]. Although the isoforms of miR-378 (miR-378a/b/c/d/e/f/g/h/i/j) are encoded by different genomic loci, they share identical seed sequences and are thus considered to have common regulatory targets [44]. In addition, a recent study by Zhu et al. suggested that miR-15b-5p mediates the autophagy of endothelial progenitor cells and influences coronary atherosclerotic heart disease via the mTOR signaling pathway [45]; however, its effect on PDK1 expression was not demonstrated previously.

Lastly, we demonstrated that IKK controlled platelet-derived exosome secretion during sepsis, which was in alignment with previous research showing that IΚΚ controls platelet secretion through regulating SNAP-23 phosphorylation [29]. SNAP-23 phosphorylation enables the formation of the soluble *N*-ethylmaleimide sensitive factor attachment protein receptors (SNAREs) complex to allow exosome release [46]. NF-κB plays a pivotal role in sepsis, and its activation is initiated by signal-induced ubiquitylation and subsequent degradation of inhibitors of kappa B (IκBs) primarily via IKK activation. There is now compelling evidence that IKK inhibition reduces multiple organ dysfunction caused by sepsis in mice [47]. However, we may be the first to show that the IKK inhibitor alleviated lung injury during sepsis through inhibiting platelet-derived exosome secretion.

## Conclusions

We demonstrated that platelet-derived exosomes containing HMGB1 and/or miRNAs induce NET formation through modulating the Akt/mTOR-related autophagy pathway. The IKK inhibitor alleviates lung injury by inhibiting platelet-derived exosome secretion. These results indicate that signals associated with exosomes released from activated platelets serve as key features of septic shock. Targeting platelet-derived exosomes may present a novel therapeutic strategy for septic shock.

## Supplementary information

**Additional file 1.** Supplementary results and methods.

## Data Availability

The datasets generated and analyzed during the current study are available from the corresponding author on reasonable request.

## Abbreviations

NET: Neutrophil extracellular trap
PMNs: Polymorphonuclear neutrophils
PBS: Phosphate buffer saline
LPS: Lipopolysaccharide
CLP: Cecal ligation and puncture
MPO: Myeloperoxidase
HMGB1: High-mobility group protein 1
miRNAs: MicroRNAs
ALI: Acute lung injury
MAP: Mean arterial pressure
ROS: Reactive oxygen species
HL-60: Human acute promyelocytic leukemia cell line
RPMI: Roswell Park Memorial Institute
FBS: Fetal bovine serum
DMSO: Dimethyl sulfoxide
RT-PCR: Real-time polymerase chain reaction
H&E: Hematoxylin and eosin
BALF: Bronchoalveolar lavage fluid
mTOR: Mammalian target of rapamycin
PI3K: Phosphatidylinositol 3-kinase
PDK1: Phosphoinositide-dependent protein kinase 1
IκBs: Inhibitors of kappa B
IKK: IκB kinase
SNAP-23: Synaptosomal-associated protein of 23 kDa
SNAREs: Soluble *N*-ethylmaleimide sensitive factor attachment protein receptors
MAPK: Mitogen-activated protein kinase
ERK: Extracellular regulated protein kinases
