# Supplementary material for: Platelet-derived exosomes promote neutrophil extracellular trap formation during septic shock
Source: Crit Care. 2020 Jun 29;24:380. doi: 10.1186/s13054-020-03082-3 (PMC7322900; doi:10.1186/s13054-020-03082-3)
Supplement: Supplementary file 1 — Additional file 1. Supplementary results and methods. [file 13054_2020_3082_MOESM1_ESM.pdf]

Supplementary Results

FIGURES

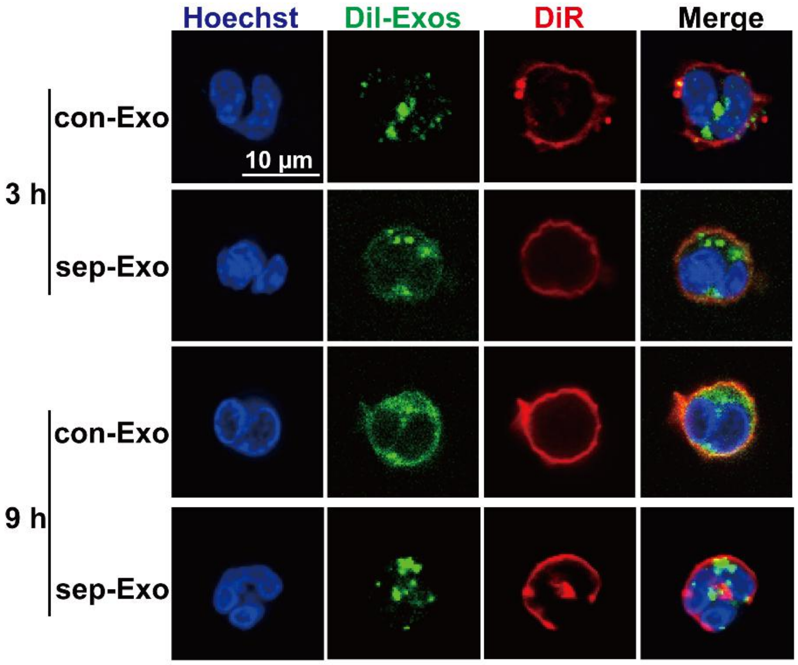

Figure S1

Supplementary Figure S1. Exosomes were isolated from the plasma of healthy volunteers (con-Exo) and septic shock patients (sep-Exo), and stained with Dil cell-labeling solutions. Immunofluorescence images show PMNs incubated with Dil-labeled exosomes (green) for 3 h or 9 h. The cell membranes of PMNs were stained with DiR (red; 5 μl/ml RPMI 1640, 30 min at 37°C; Thermo Fisher, #D127315). Nuclei were counterstained with Hoechst (blue).

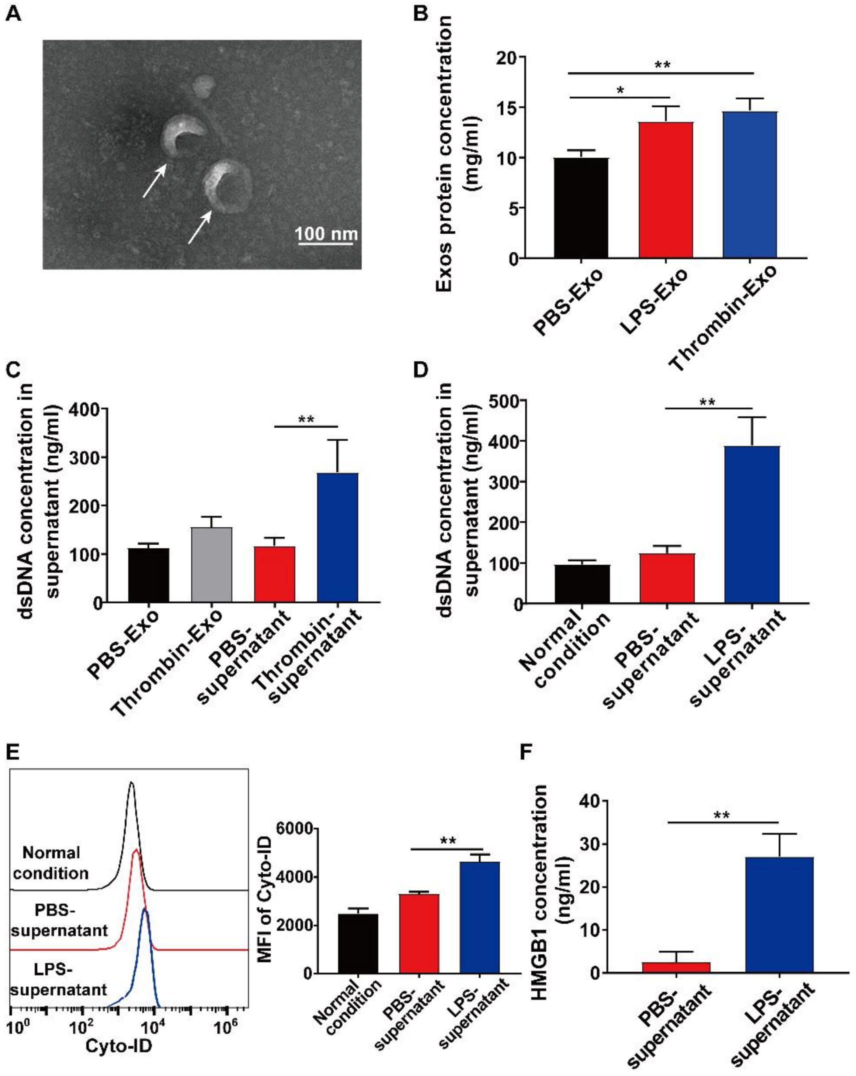

Figure S2

Supplementary Figure S2. (A-B) Platelets were stimulated with PBS or 1 μg/mL LPS or 0.1 U/mL thrombin for 3 h. Exosomes were isolated from the supernatant of stimulated platelets. (A) Electron micrograph of exosomes (indicated by white arrows) isolated from the supernatant of PBS-stimulated platelets. (B) Protein quantification of exosomes by BCA. (C) Platelets were stimulated with PBS or 1 U/mL thrombin for 3 h. Exosomes were isolated from the supernatant of stimulated platelets and cocultured with PMNs for 5 h. Exosome-free supernatants of PBS and thrombin (1 U/ml) treated platelets were collected by ultracentrifugation at 100,000g for 3 h, and

freshly isolated PMNs were cultured in exosome-free supernatant for 5 h. The supernatant dsDNA concentration was quantified by PicoGreen. (D-F) Platelets were stimulated with PBS or 1 μg/ml LPS for 3 h, and exosome-free supernatants were collected by ultracentrifugation. PMNs were cultured in normal condition (RPMI 1640 containing 10% exosomes-depleted FBS supplemented with 50 mg/mL penicillin/streptomycin) or exosome-free supernatants for 5 h. (D) The supernatant dsDNA concentration was quantified by PicoGreen. (E) PMNs autophagy was assessed by staining with Cyto-ID autophagy tracer. (F) HMGB1 concentration in exosome-free supernatant was measured by ELISA. All results are representative of 3 independent experiments. Graphs represent means ± SEM; ***P*<0.01 compared within two groups.

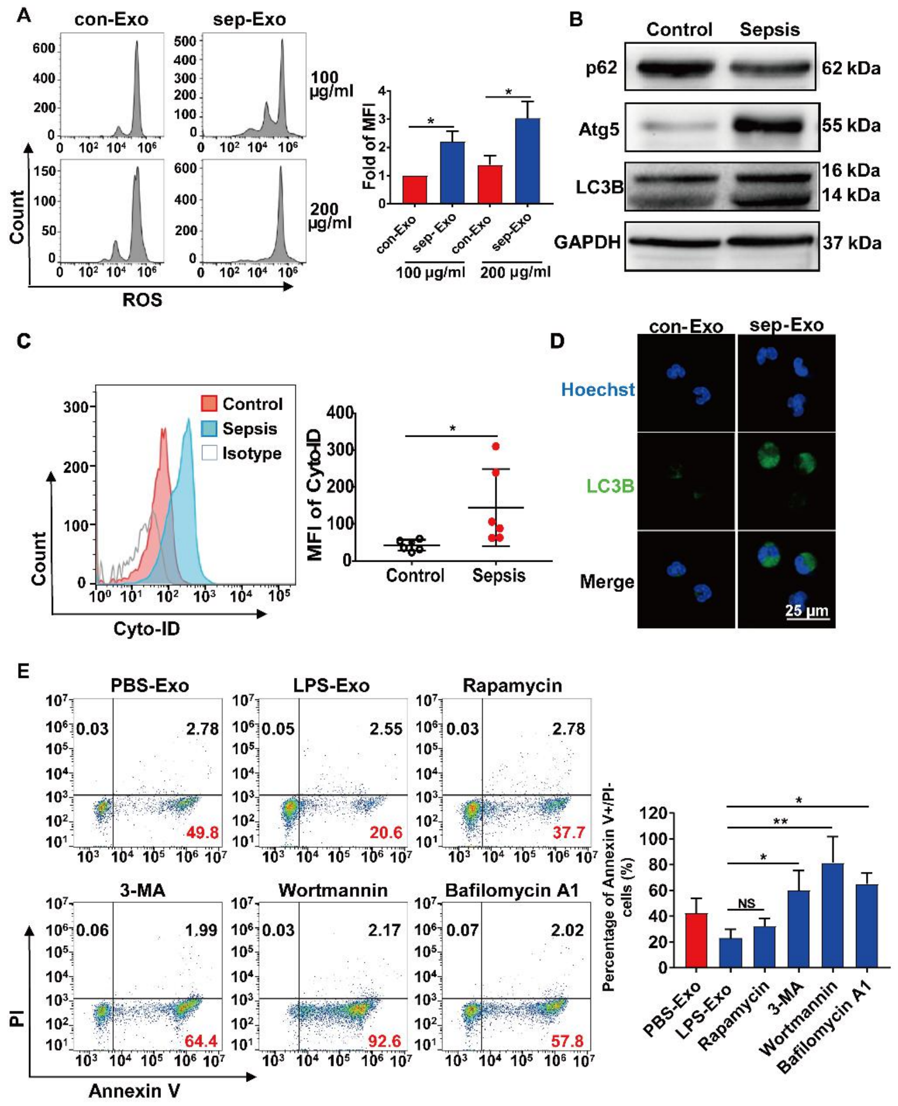

Figure S3

Supplementary Figure S3. PMNs were cocultured with exosomes isolated from the plasma of healthy controls (con-Exo) and septic shock patients (sep-Exo) or the supernatant of platelets stimulated with PBS (PBS-Exo) or LPS (LPS-Exo) for 5 h. (A) ROS expression in PMNs was measured by flow cytometry with CM-H2DCFDA staining. MFI, Mean fluorescence intensity. (B) PMNs were isolated from the peripheral blood of healthy volunteers or septic shock patients. The expression levels of p62, Atg5, and LC3B in PMNs were measured by western blot. (C) PMNs in the peripheral blood of healthy volunteers or septic shock patients were identified by

APC-CD66b staining by flow cytometry, and PMN autophagy was analyzed by staining with Cyto-ID autophagy tracer. (D) Immunofluorescence images showing LC3B expression (green) in PMNs. Nuclei were counterstained with Hoechst (blue). (E) PMNs were cocultured with PBS-Exo or LPS-Exo with or without wortmannin (150 nM), 3-MA (5 mM), bafilomycin A1 (1 μM), and rapamycin (100 nM) for 5 h. PMN death was analyzed by flow cytometry with FITC-Annexin V and PI staining. All results are representative of 3 independent experiments. Graphs represent means ± SEM; *$P<0.05$, **$P<0.01$ compared within two groups.

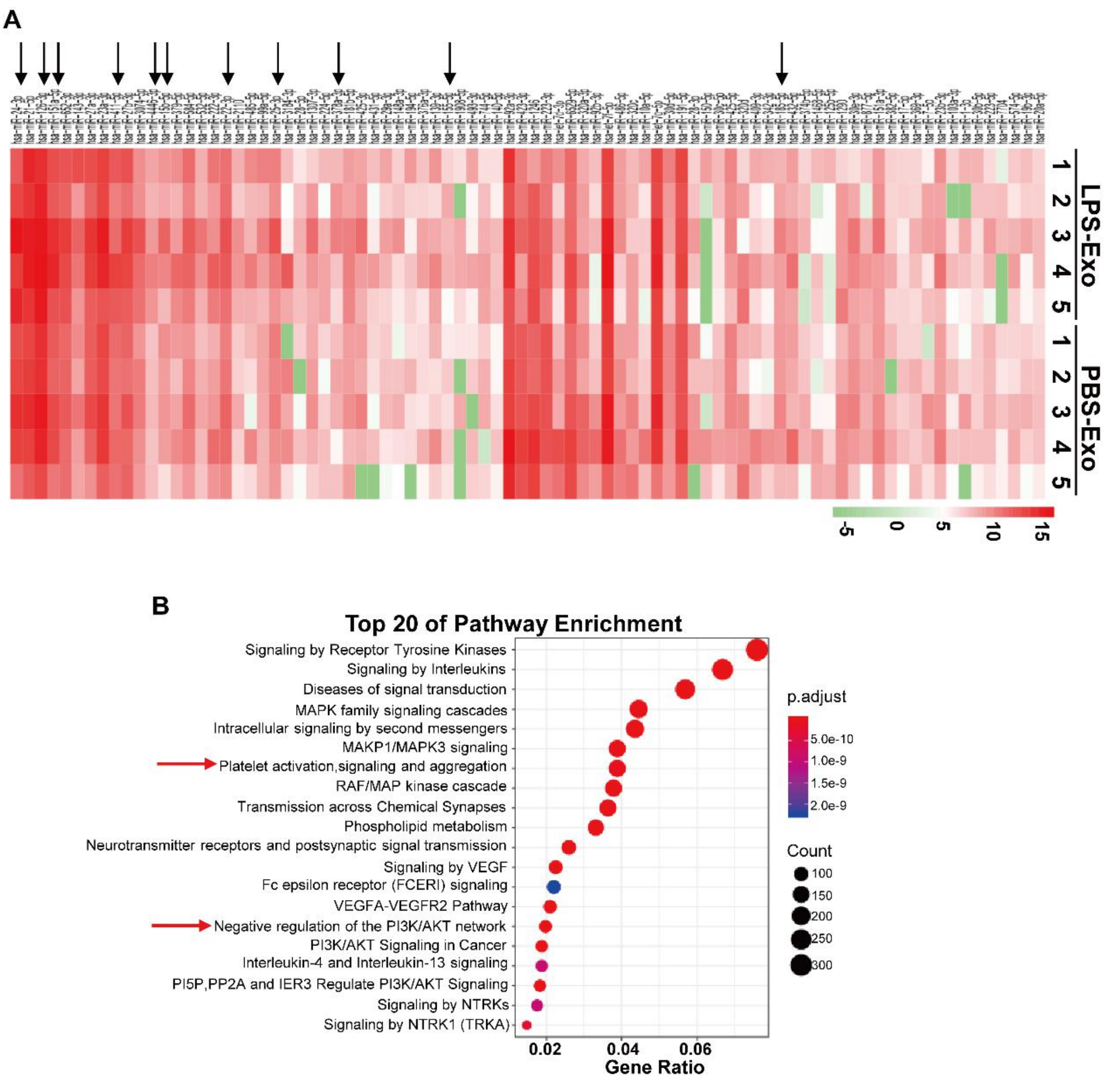

Figure S4

Supplementary Figure S4. (A) Heat map of exosomal miRNA-seq (n=5). The fluorescence intensity of 84 differentially expressed miRNAs is illustrated from high (red) to low (green). Arrows indicate miRNAs involved in negatively regulating the Akt/mTOR pathway. (B) Enriched canonical pathways regulated by differentially expressed exosomal miRNAs; the 20 most enriched pathways are shown. The gene ratio indicates the number of genes in the miRNA target list over the total genes in the respective canonical pathway.

Supplementary Materials and Methods

Reagents

The primary antibodies for cell staining and western blotting included the following: LC3B rabbit antibody (2775S), SQSTM1/p62 rabbit antibody (5114S), Atg5 (D5F5U) rabbit mAb (12994S), phospho-mTOR rabbit mAb (2983T), mTOR rabbit mAb (5536T), phospho-Akt rabbit mAb (4060S), Akt rabbit mAb (4691S), HMGB1 Rabbit mAb (6893S), histone H3 mouse mAb (14269S), and GAPDH rabbit mAb were obtained from Cell Signaling Technology (Danvers, MA, USA). The secondary antibodies Alexa Fluor 488-conjugated anti-rabbit IgG (4412S) and HRP-linked anti-rabbit IgG (7074P2) were from Cell Signaling Technology. PE anti-human CD63 antibody (353003), PE anti-human HMGB1 antibody (651404), FITC anti-human CD41 antibody (303704), and FITC anti-mouse CD41 antibody (133903) were from BioLegend (San Diego, CA, USA). Cell Death Detection ELISA (11544675001) was purchased from Roche (Indianapolis, IN, USA). The Annexin-V Detection Kit was purchased from BD Biosciences (San Jose, CA, USA). Phorbol 12-myristate 13-acetate (PMA) (P1585), N-Acetyl-L-cysteine (A7250), lipopolysaccharides from Escherichia coli O111:B4 (L2630), 3-MA (189490), and anti-MPO mAb (SAB1409321) were purchased from Sigma-Aldrich (St Louis, MO, USA). Wortmannin (HY-10197), bafilomycin A1 (HY-100558), rapamycin (HY-10219), MHY1485 (HY-B0795), BAY 11-7082 (HY-13453), and BMS-345541 (HY-10518) were purchased from MedChemExpress (Monmouth Junction, NJ, USA). CYTO-ID® Autophagy detection kit 2.0 (ENZ-KIT175-0200)

was from Enzo (Farmingdale, NY, USA). Rabbit anti-mouse thrombocyte (Platelet) (AIA31440) was from Accurate Chemical & Scientific Corporation (Westbury, NY, USA). Busulfan (A8386) was from Apexbio Corporation (Houston, TX, USA). Dil cell-labeling solutions (V22885), DiR (D12731), total exosome isolation reagent (from cell culture media) (4478359), total exosome isolation kit (from plasma) (4484450), PicoGreen assay kit (P11496), CM-H2DCFDA (C6827), and SYTOX Green (S7020) were purchased from Thermo Fisher Scientific (Waltham, MA, USA). Recombinant HMGB1 (1690-HMB-050) and recombinant human coagulation factor II/thrombin protein (1473-SE) were from R&D (Flanders, NJ, USA). BoxA from HMGB1 (HM-012) was from HMGBiotech Corporation (Milano, Italy). HMGB1 ELISA KIT (ST51011) was from IBL Corporation (Hamburg, Germany). The miRNeasy Serum/Plasma kit (217184), miScript II RT Kit (218161), and miScript SYBR Green PCR Kit (218075) were from Qiagen (Valencia, CA, USA). PrimeScript™ RT Master Mix (Perfect Real Time) (RR036A) and TB Green™ Premix Ex Taq™ (Tli RNaseH Plus) (RR420A) were from TAKARA (Tokyo, Japan).

Exosome isolation and characterization

Exosomes were isolated from the plasma of healthy controls and septic shock patients or from the supernatant of platelets stimulated ex vivo using Total Exosome Isolation Reagent. Briefly, blood was collected in centrifuge tubes containing EDTA, and the culture medium was centrifuged at 2000 g for 10 min to remove cells and debris. The supernatant was centrifuged at 10,000 g for 30 min to further remove

debris and then filtered by a 0.22 μm filter (Millipore, Burlington, MA, USA). The Total Exosome Isolation Reagents for plasma or cell culture medium, respectively, were added according to the manufacturer's instructions. The final pellet, which contained exosomes, was resuspended in PBS.

Exosome morphology was characterized using transmission electron microscopy. The size distribution of exosomes was assessed by NanoSight tracking analysis. The protein concentration of exosomes derived from 800 μL plasma of healthy controls and septic shock patients was determined by BCA. Exosome surface markers CD63 and TSG101 (tumor susceptibility gene 101) were identified by western blot loaded with equal amounts of exosomal protein (40 μg).

Flow cytometry detection of exosomes

100 μL exosomes were incubated with 10 μL aldehyde/sulfate latex beads (4 mm diameter; Thermo Fisher Scientific, Waltham, MA, USA) for 15 min at 4 °C. PBS was then added to the exosomes to reach a total volume of 400 μL, followed by incubation at 4 °C with gentle agitation overnight and centrifuged at 1000 g for 5 min. To determine the proportion of platelet-derived exosomes in the whole exosome quantity isolated from plasma, exosome-coated beads were stained with PE anti-human CD63, FITC anti-human CD41 antibody, or human IgG (Santa Cruz Biotechnology, Dallas, TX, USA) for 1 h at room temperature. To determine the expression of HMGB1 in exosomes, exosome-coated beads were stained with PE anti-human HMGB1 antibody and human IgG for 1 h at room temperature. Then, 1 mL PBS was added to wash beads twice and resuspended in 300 μL PBS.

Exosome-coated beads were gated on FSC and SSC, and analyzed by flow cytometry.

Transmission electron microscopy

After exosome isolation, the pellets were fixed with 2% glutaraldehyde in 0.1 M phosphate buffer (pH 7.4). The fixed pellets were placed on 100-mesh, carbon-coated, formvar-coated nickel grids treated with poly-L-lysine for 30 min. After washing the samples with several drops of PBS, samples were incubated with drops of buffered 1% glutaraldehyde for 5 min and then washed several times with drops of distilled water. Afterward, samples were negatively stained with drops of Millipore-filtered aqueous 4% uranyl acetate for 5 min. The stain was blotted from the grids with filter paper, and samples were allowed to dry. The microscopy images were captured by a JEOL JEM-1400 transmission electron microscope operating at 120 kV.

Platelet purification and activation

Human platelets were isolated from EDTA-anticoagulated venous blood prepared as previously described [1]. Briefly, platelet-rich plasma was obtained by centrifugation at 250 g for 10 minutes, and platelets were sedimented at 1000 g for 15 minutes and then resuspended at 10^8 platelets/mL in HEPES-Tyrode's buffer. Platelet activation was induced upon incubation with PBS or 1 μg/mL LPS or 0.1 U/mL thrombin for 3 h at 37 °C, and platelets were pelleted by centrifugation at 2000 g for 10 minutes.

Polymorphonuclear neutrophil isolation and NET induction

PMNs were isolated from the venous blood of healthy volunteers by discontinuous density gradient centrifugation with two commercially available

solutions (Histopaque-1077 and Histopaque-1119) of differential density purchased from Sigma (#10771 and #11191; St Louis, MO, USA) according to the manufacturer's instructions. The resulting cells consisted of 90% PMNs, and the viability of the isolated PMNs was 95%, as assessed by flow cytometry and Trypan blue staining, respectively. The PMNs were suspended in complete culture medium (RPMI 1640 containing 10% exosomes-depleted FBS supplemented with 50 mg/mL penicillin/streptomycin) at a concentration of 10^6 cells/mL. PMNs were treated with exosomes (100 or 200 μg/mL) in complete culture medium for up to 14 h, or 50 nM PMA for 3 h at 37 °C as positive control. In some cases, the inhibitors and enhancer used were 3-MA (5 mM), wortmannin (150 nM), bafilomycin A1 (1 μM), rapamycin (100 nM), and NAC (50 μg/mL).

HL-60 cell culture and transfection

The human acute promyelocytic leukemia cell line HL-60 (ATCC-CCL-240) was maintained in phenol red-free RPMI-1640 medium supplemented with 2 mM L-glutamine and 10% FBS in 5% CO2 at 37 °C. To allow for neutrophil-like differentiation, the cells were seeded at 10^6 cells/mL in the above-mentioned cell medium supplemented with 1.25% DMSO for 3 days, as described [2]. For transfection, cells were stimulated to undergo neutrophil-like differentiation as described above and seeded in 12-well plates (500,000 cells/well). 2 μL Lipofectamine 3000 reagent (Invitrogen, Carlsbad, CA, USA) and 2.5 μL microRNA (20 μM) control, mimics, or inhibitors (Shanghai GenePharma Co., Ltd., Shanghai, China) were allowed to form complexes in Opti-MEM medium (Thermo Fisher,

Waltham, MA, USA) for 15 min at room temperature. Subsequently, the formed complexes (150 µL) were added to the cell medium, and the neutrophil-like HL-60 cells were incubated for 24–48 h prior to analysis or coculture with exosomes.

Platelet depletion

In order to investigate the role of platelets in NET formation and ALI development, platelets were depleted as previously described [3]. Briefly, mice received 2 injections of busulfan (20 mg/kg dissolved in polyethylene glycol 400) or vehicle (poly polyethylene glycol 400) on days 0 and 3. Experiments were performed on day 14. Platelet depletion by busulfan led to 40% reduction of platelets without affecting leukocytes, which was consistent with previous study [3]. Platelet depletion was also achieved by an antibody to mouse thrombocyte serum (50 µL/mouse, i.v.) 2 h before experiments [4], which depleted circulating platelets by at least 60%.

Mouse model of CLP and in vivo exosome administration

The mouse CLP model was prepared as previously described [5]. Mice were anesthetized with ketamine (50 mg/kg) and xylazine (5 mg/kg) via i.p. injection. After disinfection, a 1 cm midline laparotomy was made in the abdomen. The cecum was then exteriorized, ligated below the cecal valve, and punctured twice with an 18-gauge needle to induce lethal sepsis. A small drop of cecal content was extruded. The cecum was then returned to the peritoneal cavity and the abdominal incision was closed with sutures. Inject 1 ml prewarmed (37 °C) normal saline *i.h.* to replace heat and hydration lost during the procedure. Place the mice under a heat lamp until their

resuscitation. No antibiotic was used after surgery. Sham animals underwent the same surgical procedures without cecum ligation and puncture.

At 24 h after surgery, the animals were euthanized by phenobarbital overdose (100 mg/kg body weight), and bronchoalveolar lavage fluid (BALF) and citrate-anticoagulated whole blood were collected at a 1:7 ratio. After BALF was centrifuged, the supernatant protein concentration was determined by BCA. In order to visualize morphological changes and NET formation in sepsis-induced ALI, lung tissue was harvested and fixed in 4% paraformaldehyde for H&E and immunofluorescence staining. H&E staining was evaluated and scored by a pathologist who was blinded to the experimental groups. To evaluate the lung injury, five independent random lung fields were evaluated per mouse for neutrophils in alveolar spaces, neutrophils in interstitial spaces, hyaline membranes, proteinaceous debris filling the airspaces, and alveolar septal thickening, and weighed according to the official American Thoracic Society workshop report on features and measurements of experimental acute lung injury in animals [6]. The resulting injury score is a continuous value between 0 and 1. For immunofluorescence staining, paraffin-embedded lung tissues were sectioned, blocked with PBS containing 1% goat serum and 3% bovine serum albumin (BSA), and then permeabilized with PBS/Triton 0.01%. Sections were incubated with MPO antibody, and then species-specific secondary antibody coupled with Alexa Fluor Dye. DNA was stained using Hoechst 33342, and sections were mounted in Vectashield Mounting Media. To determine the bacteria load, lungs were harvested at 24 h after CLP under sterile conditions. Same

amounts of the lung tissues were homogenized, incubated at 37 °C for 1 h, and centrifuged at 500 g for 5 min. The supernatant of the tissues were then properly diluted with sterile normal saline, and plated on tryptic soy agars. The agar plates were incubated at 37 °C for 24 h, and the number of bacterial colonies was calculated as colony forming units (CFU).

To explore exosome function in vivo, mice were treated with exosomes isolated from the plasma of sham or CLP mice (300 μg/mouse) through i.p. injection using 31-gauge insulin syringes. After 24 h, BALF, venous blood, and lung tissue were harvested as described previously.

Fluorescence imaging of Dil-exosomes

Exosomes were isolated from equal plasma volumes (100 μL) from sham or CLP mice, and every 10 μg isolated exosomes was incubated with 1 μL Dil labeling solution (#V22885, Thermo Fisher Scientific, Waltham, MA, USA) for 30 min at 37 °C. Then, Dil-exosomes were precipitated using Total Exosome Isolation Reagent according to the manufacturer's protocol, resuspended in 200 μL PBS buffer, and injected into mice (i.p.). After 24 h, the lungs were removed for ex vivo evaluation and the Dil-exosome biodistribution in the lungs was monitored using the IVIS Spectrum In Vivo Imaging System (PerkinElmer, Waltham, MA, USA). Filters allowing excitation at 560 nm and the collection of emission at 590 nm were used to obtain ideal images.

NET quantification assay

To quantify NETs in cell culture supernatants, plasma, and mouse BALF, we

chose the PicoGreen dsDNA Quantification Kit (Invitrogen, Carlsbad, CA, USA) and a capture ELISA based on MPO associated with DNA [7]. The PMNs were plated and cultured in 24-well plates. At the indicated time-points after treatment, 1 U/mL micrococcal nuclease (#M0247S; New England Biolabs, Ipswich, MA, USA) was added. PMNs were incubated at 37 °C for 15 min to allow the extruded DNA to detach from cell debris. Cells were then centrifuged at 1800 g for 10 min. Then, 100 μL supernatant from the cell culture medium or BALF was transferred to 96-well plates, the fluorescence activity of PicoGreen dye bound to dsDNA was excited at 480 nm, and the fluorescence emission intensity was measured at 520 nm using a spectrofluorimeter according to the manufacturer's instructions. Plasma DNA levels were also measured using the Quant-it Picogreen dsDNA Kit (Invitrogen, Carlsbad, CA, USA). Briefly, 5 μL plasma (diluted to 100 μL in TE buffer) and 100 μL Picogreen (diluted in TE buffer) were added per well in an opaque 96-well plate and read by spectrofluorimeter.

For ELISA analysis of NET concentration, 1 μg/mL anti-MPO mAb used as the capture antibody was coated onto 96-well plates overnight at 4 °C. After washing 3 times with 200 μL, 20 μL of samples was added to the wells with 80 μL incubation buffer at room temperature for 90 min. After again washing 3 times, 100 μL conjugate solution containing peroxidase-labeled anti-DNA mAb (1:10 dilution, #11544675001, Cell Death Detection ELISA; Roche, Indianapolis, IN, USA) was added. The plate was incubated for 90 min with shaking at 300 rpm at room temperature. After 3 washes, 100 μL peroxidase substrate was added. The absorbance was measured at 405

nm after incubating for 20 min at room temperature in the dark.

Flow cytometry detection of ROS and autophagy in PMNs

After coculturing with exosomes, reactive oxygen species (ROS) generation in PMNs was determined by flow cytometry with CM-H2DCFDA staining. Briefly, PMNs were removed from growth media, washed, and then resuspended in pre-warmed loading buffer containing freshly prepared probe (10 μM). After incubation for 30 min at room temperature, the fluorescence intensity was examined by flow cytometry.

Autophagy in PMNs was detected using the CYTO-ID® Autophagy Detection Kit according to the manufacturer's instructions. Red blood cells in freshly collected human blood were lysed immediately. PMNs in blood or culture medium were stained with 250 μL diluted CYTO-ID® Green dye staining solution (dilution 1:1000 in cell culture medium without phenol red indicator, supplemented with 5% FBS) at 37 °C for 30 min, followed by fixation in 4% paraformaldehyde for 10 min at room temperature. PMNs were gated on FSC and SSC, and the mean fluorescence intensity was examined by flow cytometry.

Confocal microscopy

The PMNs were allowed to settle on glass coverslips precoated with poly-L-lysine (#354085; Corning, Corning, NY, USA) for 30 min prior to coculture with exosomes for a specific period of time. Cells were washed, fixed with 4% paraformaldehyde in PBS, and blocked for 1 h prior to incubation with anti-neutrophil elastase antibody conjugated with Alexa Fluor 647 (#sc-55549 AF647; Santa Cruz

Biotechnology, Dallas, TX, USA) overnight. For SYTOX Green staining, the PMNs were incubated with 1 μM SYTOX Green reagent at 37 °C for 10 min, followed by fixation in 4% paraformaldehyde for 10 min at room temperature. Nuclei were counterstained using Hoechst 33342, and the cells were mounted in Antifade Mounting Medium (#P0126; Beyotime Biotechnology, Shanghai, China) for imaging with the FluoView™ FV1000 confocal microscope (Olympus, Tokyo, Japan).

For autophagy detection, PMNs were fixed with 4% paraformaldehyde for 10 min. After washing with PBS, the cells were permeabilized with 0.25% Triton X-100 in PBS for 10 min at room temperature, followed by blocking with 1% BSA in PBS with 0.2% Tween-20 for 1 h at room temperature to reduce nonspecific staining. The cells were then incubated with anti-LC3 antibody (1:200) at 4 °C overnight. After washing twice with PBS, the cells were incubated with Alexa Fluor 488-conjugated anti-rabbit IgG (1:500) for 1 h at room temperature. Hoechst 33342 was used to stain nuclei. The cells were then washed with PBS 3 times, followed by confocal microscopy. The mean optical density was calculated with Image J software.

Quantitative RT-PCR analysis

Total RNA was extracted from 200 μL plasma-derived exosomes using the miRNeasy Serum/Plasma Kit. RNA pellets were resuspended in 14 μL RNase-free water and quantified using Thermo ND-2000 NanoDrop. Then, 12 μL RNA solution was used for reverse transcription according to the miScript RT Kit protocol (Qiagen, Valencia, CA, USA). A miScript SYBR Green PCR Kit was used to quantify miRNA expression. Additionally, qPCR analysis was performed for miR-378a-3p and

miR-15b-5p expression in cells. Briefly, RNA was extracted by TRIzol reagent (15596026; Invitrogen, Carlsbad, CA, USA), and qRT-PCR was conducted using the Mir-X miRNA qRT-PCR SYBR Kit (#638316; TaKaRa, Tokyo, Japan). The relative expression was calculated using the comparative cycle threshold (Ct) method ($2^{-\Delta\Delta CT}$) normalized to U6. The miRNA qPCR primers were purchased from Guangzhou RiboBio Corporation (Guangzhou, China).

Luciferase assay

The 3′-UTR of the PDK1 sequence containing the predicted miR-378a-3p and miR-15b-5p binding sites and its mutant were cloned into the plasmid vector and transfected into HEK293 cells. In all transfections, a Renilla luciferase vector was co-transfected to monitor transfection efficiency. All luciferase results were reported as relative light units: the average of the observed photinus pyralis firefly activity divided by the average of the activity recorded from the Renilla luciferase vector.

Sequencing of miRNA

Total RNA was extracted from exosomes using the miRNeasy Serum/Plasma Kit according to the manufacturer's instructions. Subsequently, total RNA was qualified and quantified using a NanoDrop and Agilent 2100 bioanalyzer (Thermo Fisher Scientific, Waltham, MA, USA). A library was prepared with 1 μg total RNA for each sample. Total RNA was purified by electrophoretic separation on a 15% urea denaturing polyacrylamide gel electrophoresis (PAGE) gel, and small RNA regions corresponding to the 18-30 nt bands in the marker lane (14-30 ssRNA Ladder Marker, TAKARA, Tokyo, Japan) were excised and recovered. Then, the 18-30 nt small

RNAs were ligated to adenylated 3′ adapters that were annealed to unique barcodes, followed by the ligation of 5′ adapters. The adapter-ligated small RNAs were subsequently transcribed into cDNA by SuperScript II Reverse Transcriptase (Invitrogen, Carlsbad, CA, USA), and then several rounds of PCR amplification with PCR Primer Cocktail and PCR Mix were performed to enrich the cDNA fragments. The PCR products were selected by agarose gel electrophoresis with approximately 110-130 bp target fragments and then purified by the QIAquick Gel Extraction Kit (Qiagen, Valencia, CA, USA). The library was examined qualitatively and quantitatively in two methods: the fragment size distribution was verified using the Agilent 2100 bioanalyzer, and the library was quantified using real-time quantitative PCR (qPCR) (TaqMan Probe). The final ligated PCR products were sequenced using the BGISEQ-500 platform (BGI Group, Shenzhen, China).

References

1. Zeng Z, Xia L, Fan X, Ostriker AC, Yarovinsky T, Su M, Zhang Y, Peng X, Xie Y, Pi L, Gu X, Chung SK, Martin KA, Liu R, Hwa J, Tang WH. Platelet-derived miR-223 promotes a phenotypic switch in arterial injury repair. J Clin Invest. 2019; 129(3):1372-1386.
2. Khandagale A, Lazzaretto B, Carlsson G, Sundin M, Shafeeq S, Römling U, Fadeel B. JAGN1 is required for fungal killing in neutrophil extracellular traps: Implications for severe congenital neutropenia. J Leukoc Biol. 2018; 104(6):1199-1213.
3. Zarbock A, Singbartl K, Ley K. Complete reversal of acid-induced acute lung injury by blocking of platelet-neutrophil aggregation. J Clin Invest. 2006; 116(12):3211-3219.
4. Clark SR, Ma AC, Tavener SA, McDonald B, Goodarzi Z, Kelly MM, Patel KD, Chakrabarti S, McAvoy E, Sinclair GD, Keys EM, Allen-Vercoe E, Devinney R, Doig CJ, Green FH, Kubes P. Platelet TLR4 activates neutrophil extracellular traps to ensnare bacteria in septic blood. Nature medicine. 2007; 13(4):463-469.
5. Chen L, Zhao Y, Lai D, Zhang P, Yang Y, Li Y, Fei K, Jiang G, Fan J. Neutrophil extracellular traps promote macrophage pyroptosis in sepsis. Cell Death Dis. 2018;

9(6):597.

6. Matute-Bello G, Downey G, Moore BB, Groshong SD, Matthay MA, Slutsky AS, Kuebler WM. An official American Thoracic Society workshop report: features and measurements of experimental acute lung injury in animals. Am J Respir Cell Mol Biol. 2011; 44(5):725-738.
7. Caudrillier A, Kessenbrock K, Gilliss BM, Nguyen JX, Marques MB, Monestier M, Toy P, Werb Z, Looney MR. Platelets induce neutrophil extracellular traps in transfusion-related acute lung injury. J Clin Invest. 2012; 122(7):2661-2671.
